# Transportation price, product differentiation, and R&D in an oligopoly

**DOI:** 10.1371/journal.pone.0273904

**Published:** 2022-09-09

**Authors:** Daishoku Kanehara, Keita Kamei

**Affiliations:** 1 Faculty of Economics, Toyo University, Tokyo, Japan; 2 Department of Economics, Seinan Gakuin University, Fukuoka, Japan; University of Oklahama Norman Campus: The University of Oklahoma, UNITED STATES

## Abstract

In this article, we construct an international oligopoly that explicitly incorporates transporter behavior. In each country, there is one firm that produces differentiated goods and invests in product-differentiating R&D and one transporter that transports the differentiated goods. We adopt a three-stage game in which the firms decide their R&D investment level to determine the degree of horizontal differentiation, the transporters determine the transportation prices through Cournot competition, and then the firms determine the quantities of production. We find that an increase in R&D efficiency in the product differentiation of firms leads to a decrease in transportation prices. We also reveal that an increase in the efficiency of product differentiation always reduces the profits of firms. These results explain the empirically plausible long-term trend of declining transportation prices and also provide a counterintuitive implication that efficiency gains reduce the degree of product differentiation.

## Introduction

Transportation prices have generally been treated as a key factor in international trade. In a series of new trade theories, [1] revealed that a reduction in trade costs (including transportation prices) promoted the international trade of differentiated goods. In fact, as [2] mentioned, with the expansion of world trade, time series have shown a gradual decline in the air transportation price index. Many studies on new trade theory assume trade costs to be an exogenous variable, so they do not investigate how product differentiation affects trade costs (i.e, transportation prices). An example of this can be seen within Maserati—the luxury car brand—which transports its latest models by air rather than by sea. (See the following website: https://www.airbridgecargo.com/en/news/228/airbridgecargo-provided-dedicated-lift-for-28-maserati-granturismo-cars.) In addition, [3] have shown that transportation prices are endogenously determined by transportation firms. They reveal that the larger the degree of product differentiation is—that is, the smaller the price elasticity of (import) demand—the higher the prices imposed by transporters.

In this article, we construct an international oligopoly model with endogenous product differentiation (product R&D à la [4]) and endogenous transportation prices. Our main conclusion is that when product differentiation is more efficient, R&D investment in product differentiation, transportation prices, and the profits of the differentiated good firm decrease monotonically. The intuition for this is that higher efficiency of differentiation leads to lower R&D investment, which encourages lower differentiation. This in turn decreases marginal revenue in foreign markets, thereby lowering the amount of exports. This effect is found to lower transportation prices through Cournot competition among transporters. Furthermore, a reduction in the efficiency of differentiation always lowers the total revenue of a firm while also lowering its total cost. Especially in the setting of this paper, the total revenue reduction always dominates the cost reduction effect. Thus, the reduction in the efficiency of differentiation reduces the profits of differentiated goods firms.

In recent years, there have been many studies on endogenous transportation prices. Notable examples include [5–11]. In addition, [12, 13] incorporated cost-reducing R&D into an international oligopoly with endogenous transportation prices. Conversely, our model focuses on product R&D (i.e., horizontal product differentiation in an international oligopoly with endogenous transportation prices). [14] also incorporate the relationship between product differentiation and transportation prices, However, their main motivation is the consumer surplus, and they do not focus on the effect of the efficiency of product differentiation on transportation prices.

Because [15] noted out that the proportion of R&D on variety is lower than that on quality improvements and [16] show that cost-reducing R&D and quality improvement are equivalent in a general representative firm economy, it seems natural to focus on the effect of cost-reducing R&D on endogenous transportation prices. However, recent developments in information and communications technology (ICT) enable us to endogenize the location of production. Studies on location choice are systematically summarized in [17]. Although this phenomenon can reduce the importance of the transportation of products in horizontal product differentiation, the production location itself can be a key factor in such differentiation. Thus, the effect of product R&D on endogenous transportation prices remains important regardless of recent ICT developments.

This article also relates to oligopolistic competition and product R&D. [4, 18] constructed an oligopolistic competition model under both product and process R&D. They employed the utility function of [19] and derived the equilibrium not only under Bertrand competition but also under Cournot competition. [20] extended the model in [4] to an oligopolistic general equilibrium and investigated how trade liberalization affects product R&D. They revealed that protection from international trade (an increase in the number of shielded sectors) has ambiguous effects on welfare. Furthermore, [21] showed that additional product differentiation increases trade benefits.

To the best of our knowledge, there are few studies on the relationship between the degree of product differentiation and transportation prices. This implies that we do not know how the degree of differentiation (or quality of products) affects transportation prices through strategic interaction. This is partly because [4] and their successors did not derive the closed-form solution for product R&D. Therefore, we have constructed a tractable framework that incorporates both product R&D and endogenous transportation prices and reveals the relationship among the efficiency of product differentiation, the degree of differentiation and transportation prices, which is consistent with the process R&D case in [12]. On the other hand, the efficiency of product differentiation positively affects the equilibrium profits of firms in a differentiated product sector. In the above analysis, we make a the strong assumption on the cost function of R&D, but we numerically derive the same result under a quadratic cost function.

This article is composed of the following sections: Section 2 constructs the basic model, Section 3 solves the model, Section 4 provides the main results, Section 5 numerically verifies the main result under the usual assumption on the cost function, and Section 6 concludes the article.

## The model

We assume two perfectly symmetric countries (country one and country two) in an open economy. This implies that the analysis of one country can be applied to the other. In this economy, there are two sectors: a differentiated product sector and a homogenous product sector. Homogenous products are nontradable, and producers in this sector face perfect competition. In contrast, differentiated products are tradable, and each country has one identical firm that produces its own variety. For simplicity, the marginal cost of differentiated products is assumed to be zero. We denote the quantity of the differentiated goods produced in country *i* and supplied to country *j* by *q*_*ij*_. To transport one unit of product from one country to another, the firm is required to pay a transportation price *f* to symmetric transporters located in each country. The determination of *f* is discussed later in this section. To reduce competition, firms can choose varieties through their product R&D investment *d*_*i*_. As previously discussed, the profit of a firm in country *i* becomes

$$\begin{array}{c} \pi_{i}=p_{ii}q_{ii}+p_{ij}q_{ij}-fq_{ij}-c(d_{i}), \end{array}$$ (1)

where *p*_*ij*_ is the price of firm *i*’s product in country *j* and *c*(*d*_*i*_) is the cost of R&D when the investment level is *d*_*i*_.

We also assume that consumers are perfectly symmetric. The utility function of consumers in country *i* is defined by

$$\begin{array}{c} u_{i}=a(q_{ii}+q_{ji})-\frac{1}{2}[q_{ii}^{2}+q_{ji}^{2}]-sq_{ii}q_{ji}+m_{i}, \end{array}$$ (2)

where *a*(> 0) is a parameter, *m*_*i*_ is the quantity of the homogenous goods, and *s* (0 < *s* < 1) is the inverse of the degree of differentiation between two products. The budget constraint of consumers in country *i* becomes

$$\begin{array}{c} p_{ii}q_{ii}+p_{ji}q_{ji}+m_{i}=I_{i}. \end{array}$$ (3)

The utility maximization of both countries implies the following inverse demands

$$\begin{array}{ccc} p_{ii} & = & a-q_{ii}-sq_{ji}, \end{array}$$ (4)

$$\begin{array}{ccc} p_{ij} & = & a-q_{ij}-sq_{jj}. \end{array}$$ (5)

These indicate that higher differentiation (i.e., *s* is low) implies higher prices *p*_*ii*_, *p*_*ij*_.

We assume that the degree of differentiation is determined by the relatively low R&D investment level of both firms:

$$\begin{array}{c} s≔1-\text{min}\left\{d_{1},d_{2}\right\},0<d_{1},d_{2}<1. \end{array}$$ (6)

The R&D cost function *c*(*d*_*i*_, *e*) has the following characteristics: $c(0,e)=0,\frac{dc(d_{i},e)}{dd_{i}}>0,\frac{d^{2}c(d_{i},e)}{dd_{i}^{2}}>0,\frac{dc(d_{i},e)}{de}<0$, where *e* (> 0) is the efficiency of product differentiation. In this article, we assume that each industry has identical efficiency *e* in differentiation. The efficiency of product differentiation *e* refers to the ease with which an industry or each good can be differentiated by nature, and it can be interpreted that standardized goods (such as industrial chemical products) are difficult to differentiate. In contrast, in the case of nonstandardized goods (e.g., luxury cars and ornaments), horizontal differentiation is easy, and the efficiency of product differentiation is high.

Finally, we assume that the marginal cost of transportation for transporters is zero and that there are two symmetric transporters (*i* = 1.2) that choose the amounts of transportation *χ*_*i*_ to maximize their profits

$$\begin{array}{c} \pi_{i}^{f}=f_{i}\chi_{i}, \end{array}$$

where *f*_*i*_ is the international transportation price of transporter *i* and *χ*_1_ + *χ*_2_ = *q*_12_ + *q*_21_. This *f*_*i*_ satisfies *f*_*i*_ = *f* in equilibrium under Cournot competition in Step 2.

## Analysis

We assume that the decision-making process between the transporter and the differentiated firms is the following three-stage game: In step 1, firms decide their R&D investment level *d*_*i*_ to maximize their profits. In step 2, duopolistic transporters maximize their profits under Cournot competition, and thus, the equilibrium transportation price *f* is determined by this process. In step 3, based on the results, firms produce their differentiated products under Cournot competition.

### Step 3

Based on the inverse of the degree of differentiation *s* and the transportation price *f*, firm *i* chooses the quantities to be supplied to home *q*_*ii*_ and foreign *q*_*ij*_ to maximize its profit:

$$\begin{array}{c} \pi_{i}=p_{ii}q_{ii}+p_{ij}q_{ij}-fq_{ij}-c(d_{i}). \end{array}$$

By the first-order conditions and inverse demand functions Eqs (4) and (5), the optimal quantities become

$$\begin{array}{c} q_{ii}=\frac{(2-s)a+sf}{4-s^{2}},q_{ij}=\frac{(2-s)a-2f}{4-s^{2}}. \end{array}$$ (7)

It is evident that a higher transportation price *f* has a positive effect on domestic supply *q*_*ii*_ and a negative effect on exports *q*_*ij*_. This is because if the transportation price *f* is high, it impedes exports and mitigates competition between home and foreign firms.

By Eq (7), the effect of the inverse of the degree of differentiation *s* on the quantities is

$$\begin{array}{c} \frac{\partial q_{ii}}{\partial s}=\frac{-a{\left(s-2\right)}^{2}+f\left(4+s^{2}\right)}{{\left(s^{2}-4\right)}^{2}}<0,\frac{\partial q_{ij}}{\partial s}=-\frac{a{\left(s-2\right)}^{2}+4fs}{{\left(s^{2}-4\right)}^{2}}<0. \end{array}$$

Although first inequality might not be intuitive, since we denote the inverse of the degree of differentiation by *s*, this inequality implies that the increase in the inverse of the degree of differentiation (that is, an increase in *s*) on *q*_*ii*_ is negative. The intuition behind this is that lower levels of differentiation intensify Cournot competition and reduce production. On the other hand, a decrease in the level of differentiation promotes a decrease in marginal profits from exports and reduces exports by foreign firms. This decline in exports increases marginal profits in the home market and increases home output by domestic firms.

### Step 2

We assume that to export differentiated products, firms are required to pay the unit transportation price *f* to transporters (being symmetric, the transporters will exhibit the same transportation price). Additionally, we assume that the marginal cost of transportation is zero and each country has a transporter that can transport differentiated products regardless of the destination. The duopolistic (and symmetric) transporters choose the amounts of transportation *χ*_*i*_ under Cournot competition. The profit of the transporter in country *i* $\pi_{i}^{f}$ is $\pi_{i}^{f}=f_{i}\chi_{i}$, where *χ*_*i*_ + *χ*_*j*_ = *q*_*ij*_ + *q*_*ji*_. By symmetry, *χ*_*i*_ = *q*_*ij*_ is satisfied in equilibrium. Based on Eq (7) and the symmetric property in differentiated products, the inverse demand is

$$\begin{array}{c} f_{i}=\frac{a(2-s)}{2}-\frac{\chi_{i}+\chi_{j}}{4}(4-s^{2}). \end{array}$$

The objective function of the maximization problem of the transporter in country *i* $\pi_{i}^{f}$ becomes

$$\begin{array}{c} \pi_{i}^{f}=[\frac{a(2-s)}{2}-\frac{\chi_{i}+\chi_{j}}{4}(4-s^{2})]\chi_{i}. \end{array}$$

The first-order condition for profit maximization becomes

$$\begin{array}{ccc} \frac{\partial \pi_{i}^{f}}{\partial \chi_{i}} & = & \frac{2a(2-s)-\left(4-s^{2}\right)\left(2\chi_{i}+\chi_{j}\right)}{4}=0. \end{array}$$

The first-order condition for the transporter in country *j* also becomes

$$\begin{array}{ccc} \frac{\partial \pi_{j}^{f}}{\partial \chi_{j}} & = & \frac{2a(2-s)-\left(4-s^{2}\right)\left(2\chi_{j}+\chi_{i}\right)}{4}=0. \end{array}$$

By the first-order conditions, the optimal amounts of transportation *χ*_*i*_, *χ*_*j*_ are

$$\begin{array}{c} \chi_{i}=\chi_{j}=\frac{2a}{3(2+s)} \end{array}$$ (8)

Therefore, the optimal transportation price of transporters *i* and *j*, *f*_*i*_*f*_*j*_ becomes

$$\begin{array}{c} f_{i}=f_{j}=\frac{(2-s)a}{6}. \end{array}$$

Since *f*_*i*_ = *f*_*j*_, there exists an equilibrium market transportation price *f* that satisfies

$$\begin{array}{c} f=\frac{(2-s)a}{6}. \end{array}$$ (9)

This implies that a higher level of differentiation increases the transportation price by reducing competition.

To summarize Eqs (7), (8) and (9), *q*_*ii*_ and *q*_*ij*_ become

$$\begin{array}{c} p_{ii}=\frac{a(6+s)}{6(2+s)},p_{ij}=\frac{a(8-s^{2})}{6(2+s)},q_{ii}=\frac{a(6+s)}{6(2+s)},q_{ij}=\frac{2a}{3(2+s)}. \end{array}$$ (10)

### Step 1

Using the previous discussion, firm *i* chooses product R&D investment *d*_*i*_ to maximize *π*_*i*_. Since firms are symmetric and the degree of differentiation is specified as *s* = 1 − min{*d*_1_, *d*_2_}, becomes *s* = 1 − *d*_*i*_. To simplify the notation, we denote *p*_*ii*_*q*_*ii*_ + *p*_*ij*_*q*_*ij*_ − *fq*_*ij*_ by *Π*_*i*_. The first-order condition in Step 1 becomes

$$\begin{array}{cc} \frac{\partial \pi_{i}}{\partial d_{i}}=0 & \Leftrightarrow \frac{\partial c(d_{i},e)}{\partial d_{i}}=\frac{\partial \Pi_{i}}{\partial d_{i}}\\ & \Leftrightarrow \frac{\partial c(d_{i},e)}{\partial d_{i}}=\frac{2a^{2}(11-d_{i})}{9{\left(3-d_{i}\right)}^{3}}. \end{array}$$ (11)

To make the analysis tractable and derive a closed-form solution, we specify the R&D cost function *c*(*d*_*i*_, *e*) as the following Assumption 1.

**Assumption 1**

$$\begin{array}{c} c(d_{i},e)≔\frac{1}{e}(\frac{1}{3-d_{i}}-\frac{1}{3}). \end{array}$$ (12)

This specification might appear strong, but, *c*(*d*_*i*_, *e*) satisfies desirable conditions such as convexity on *d*_*i*_: $c(0,e)=0,\frac{\partial c(d_{i},e)}{\partial d_{i}}>0,\frac{\partial^{2}c(d_{i},e)}{\partial d_{i}^{2}}>0,\frac{\partial c(d_{i},e)}{\partial e}<0$. Additionally, in contrast to [4], who do not specify the R&D cost function and thus cannot derive a closed-form solution, our result is fundamentally consistent with [12] except for the effect of the efficiency of differentiation *e* on the equilibrium profit $\pi_{i}^{*}$; our specification implies no loss of generality. Additionally, we numerically verify the result of our comparative statistics under relatively high efficiency of differentiation in Section 5. Under this assumption, Eq (11) becomes

$$\begin{array}{c} 9(3-d_{i})=a^{2}e(11-d_{i}). \end{array}$$

Thus, the optimal product R&D investment $d_{i}^{*}$ is

$$\begin{array}{c} d_{i}^{*}=\frac{11a^{2}e-27}{a^{2}e-9}, \end{array}$$ (13)

and the R&D cost in equilibrium c* becomes

$$\begin{array}{c} c^{*}(d_{i})=\frac{27-11a^{2}e}{12a^{2}e^{2}}. \end{array}$$ (14)

## Comparative statistics

Based on Eq (13), we derive

$$\begin{array}{c} \frac{\partial d_{i}^{*}}{\partial e}=-\frac{72a^{2}}{{\left(a^{2}e-9\right)}^{2}}<0. \end{array}$$ (15)

Thus, the following Proposition 1 is derived:

**Proposition 1** *Higher cost efficiency in R&D investment e leads to lower levels of R&D investment d*_*i*_.

Proposition 1 also implies that

$$\begin{array}{c} \frac{\partial c^{*}(d_{i})}{\partial e}=\frac{11a^{2}-54}{24a^{2}e^{3}}<0. \end{array}$$

Proposition 1 appears to offer the counterintuitive result that the higher the efficiency of product differentiation *e*, the lower the level of product R&D investment *d*_*i*_. The intuition behind Proposition 1 is as follows: in Step 1, the degree of differentiation *s* has a negative effect on quantities of the production *q*_*ii*_, *q*_*ij*_. This effect is explained as follows: the higher the degree of differentiation *s* is, the greater the intensity of Cournot competition. This is because the higher the degree of differentiation *s* is, the more advantageous it is for the foreign firm, which is required to pay the transportation price *f* to export. Furthermore, Step 2 shows that the degree of differentiation *s* has the effect of reducing the transportation price *f*. This decrease in the transportation price *f* leads to a further intensification of Cournot competition. Given these effects of the degree of differentiation *s* on competition, to avoid competition, firms reduce the level of R&D investment *d*_*i*_.

Additionally, using Eq (9), the optimal transportation price f* becomes

$$\begin{array}{c} f^{*}=\frac{2a(a^{2}e-3)}{a^{2}e-9}, \end{array}$$

and the effect of a high *e* on the transportation price f* also becomes

$$\begin{array}{c} \frac{\partial f^{*}}{\partial e}=-\frac{12a^{3}}{{\left(a^{2}e-9\right)}^{2}}<0. \end{array}$$

Moreover, we obtain s*, $q_{ii}^{*}$, and $q_{ij}^{*}$ in equilibrium as follows:

$$\begin{array}{c} s^{*}=\frac{-10a^{2}e+18}{a^{2}e-9},q_{ii}^{*}=\frac{a}{12}+\frac{3}{4ae},q_{ij}^{*}=-\frac{a}{12}+\frac{3}{4ae}. \end{array}$$

Similarly, $p_{ii}^{*}$ and $p_{ij}^{*}$ become

$$\begin{array}{c} p_{ii}^{*}=\frac{a}{12}+\frac{3}{4ae},p_{ij}^{*}=\frac{23a^{4}e^{2}-54a^{2}e-81}{12a^{3}e^{2}-108ae}. \end{array}$$

$\frac{9}{5}\leq a^{2}e\leq \frac{27}{11}$ guarantees that $d_{i}^{*}$ and s* should be in [0, 1], and this also guarantees $f^{*}\geq ,q_{ij}^{*}\geq 0,p_{ij}^{*}\geq 0$,. Thus, when

$$\begin{array}{c} \frac{9}{5}\leq a^{2}e\leq \frac{27}{11} \end{array}$$ (16)

is satisfied, there is a well-defined unique equilibrium. Hereafter, we assume this condition. Using the optimal transportation price f*, the following Proposition 2 is derived naturally:

**Proposition 2** Higher cost efficiency in R&D investment e leads to lower levels of the optimal transportation price f*.

An example of Proposition 2 is observed in the luxury car industry, as described in the introduction. The intuition behind Proposition 2 is closely related to the intuition behind Proposition 1. Proposition 2 suggests that the higher the efficiency of differentiation *e* is, the lower the optimal transportation price f*. This is because the degree of differentiation *s* intensifies Cournot competition, which increases exports, and in response, transporters lower the transportation price to increase quantity of exports. This also contributes to Proposition 1, which states that the higher the efficiency of differentiation is, the lower the level of product R&D investment.

Although there is a difference in the types of R&D (product and process R&D), Proposition 1 and Proposition 2 and the intuition for them are similar to the results in [12]. On the other hand, different from [12], we derive Proposition 3 to characterize profits.

**Proposition 3** *Higher cost efficiency in R&D investment e leads to lower levels of equilibrium profit for firm i* $\pi_{i}^{*}$ .

Proposition 3 is derived by the equilibrium profit of firm *i*

$$\begin{array}{c} \pi_{i}^{*}=\frac{a^{2}e+33}{72e}, \end{array}$$ (17)

where

$$\begin{array}{c} \frac{\partial \pi_{i}^{*}}{\partial e}=-\frac{11}{24e^{2}}<0 \end{array}$$ (18)

is satisfied. Thus, high efficiency *e* in differentiation negatively affects the profit of firms *π*_*i*_.

To understand the intuition behind this result, we derive the effect of *e* on *p*_*ii*_, *p*_*ij*_, *q*_*ii*_, *q*_*ij*_ as follows:

$$\begin{array}{ccc} \frac{\partial p_{ii}^{*}}{\partial e}=-\frac{3}{4ae^{2}} & < & 0,\\ \frac{\partial p_{ij}^{*}}{\partial e}=-\frac{3(17a^{4}e^{2}-18a^{2}e+81)}{4ae^{2}{\left(a^{2}e-9\right)}^{2}} & < & 0,\\ \frac{\partial q_{ii}^{*}}{\partial e}=-\frac{3}{4ae^{2}} & < & 0,\\ \frac{\partial q_{ij}^{*}}{\partial e}=-\frac{3}{4ae^{2}} & < & 0. \end{array}$$

Thus, we decompose the effect of *e* on $\pi_{i}^{*}$ as follows:

$$\begin{array}{c} \frac{d\pi_{i}^{*}}{de}=(\underset{\left(-\right)}{\underset{︸}{\frac{\partial p_{ii}}{\partial e}}}\cdot q_{ii}+\underset{\left(-\right)}{\underset{︸}{\frac{\partial q_{ii}}{\partial e}}}\cdot p_{ii}+\underset{\left(-\right)}{\underset{︸}{\frac{\partial p_{ij}}{\partial e}}}\cdot q_{ij}+\underset{\left(-\right)}{\underset{︸}{\frac{\partial q_{ij}}{\partial e}}}\cdot p_{ij})-(\underset{\left(-\right)}{\underset{︸}{\frac{\partial f}{\partial e}}}\cdot q_{ij}+\underset{\left(-\right)}{\underset{︸}{\frac{\partial q_{ij}}{\partial e}}}\cdot f+\underset{\left(-\right)}{\underset{︸}{\frac{\partial c(d_{i})}{\partial e}}}). \end{array}$$ (19)

Additionally, we segment the equilibrium profit of firm *i* into four parts: profit from domestic supply (PDS) *p*_*ii*_*q*_*ii*_, profit from exports (PEX) *p*_*ij*_*q*_*ij*_, transport charge (TC) *fq*_*ij*_, and R&D cost *c*(*d*_*i*_)

$$\begin{array}{ccc} \frac{\partial PDS}{\partial e}=-\frac{9+a^{2}e}{8a^{2}e^{3}} & < & 0,\\ \frac{\partial PEX}{\partial e}=-\frac{9}{8a^{2}e^{3}}-\frac{23a}{72} & < & 0,\\ \frac{\partial TC}{\partial e}=-\frac{1}{2e^{2}} & < & 0,\\ \frac{\partial R\&DCost}{\partial e}=\frac{11a^{2}e-54}{24a^{2}e^{3}} & < & 0. \end{array}$$

The above results show that an increase in *e*, i.e., an increase in the efficiency of R&D, always decreases both total revenue (PDS + PEX) and total cost(TC + R&D cost). An increase in the efficiency of R&D always reduces R&D investment, as stated in Proposition 1. This encourages competition among firms and causes the total revenue to fall. This competition effect also reduces the total cost. On the other hand, an increase in *e* has a direct negative effect on the R&D cost by improving R&D efficiency. Using Eq (12), this direct effect can be observed in $\frac{\partial c(d_{i},e)}{\partial d_{i}}=-\frac{d_{i}}{3(3-d_{i})e}<0.$ To compare both the competitive and direct effects on the equilibrium profit, the effect of decreasing total revenue by competition always exceeds the reduction of the total cost by the two effects. The main difference between our results and those in [12] lies in this Proposition 3. Their result is that the effect of efficiency *e* on the equilibrium profit can be positive when efficiency *e* is relatively low.

Using Proposition 1 and Proposition 3, $\frac{\partial \pi_{i}^{*}}{\partial d_{i}}>$ is derived naturally. This implies that the effort of firm *i* on differentiation, which is positive in a well-defined unique equilibrium, should always increase its profit. Thus, there is no prisoners’ dilemma between firms in endogenous product differentiation.

The above analysis essentially focuses on the effect of the efficiency of product differentiation *e* on the endogenous variables $f^{*},p_{ii}^{*},p_{ij}^{*},q_{ii}^{*},q_{ij}^{*},\pi_{i}^{*}$. As a corollary of this result, using Eq (15), we can easily derive the effect of the inverse of the degree of differentiation *s* on the endogenous variables as follows:

$$\begin{array}{c} \frac{\partial f^{*}}{\partial s}>0,\frac{\partial p_{ii}^{*}}{\partial s}>0,\frac{\partial p_{ij}^{*}}{\partial s}>0,\frac{\partial q_{ii}^{*}}{\partial s}>0,\frac{\partial q_{ij}^{*}}{\partial s}>0,\frac{\partial \pi_{i}^{*}}{\partial s}>0. \end{array}$$ (20)

Regarding the trade promotion effect, we examine how the inverse of the degree of differentiation *s* affects the inter/intra ratio $\frac{q_{ij}^{*}}{q_{ii}^{*}}$, which represents the degree of dependence on foreign trade. From the above discussion, the ratio $\frac{q_{ij}^{*}}{q_{ii}^{*}}$ becomes

$$\begin{array}{c} \frac{q_{ij}^{*}}{q_{ii}^{*}}=\frac{9{\left(2+s\right)}^{2}}{a^{2}(6+s)}. \end{array}$$

Using this,

$$\begin{array}{c} \frac{d\frac{q_{ij}^{*}}{q_{ii}^{*}}}{ds}=\frac{9(s+10)(s+2)}{a^{2}{\left(6+s\right)}^{2}}>0. \end{array}$$ (21)

Thus, the inverse of the degree of differentiation *s* is positively associated with trade. In contrast to conventional wisdom, this result also implies that the efficiency of product differentiation *e* negatively affects trade.

The specification of the R&D cost function and the analytical comparative statistics in Section 4 cannot be applied to the monopolistic transporter case discussed in [12] because the well-definedness of the optimal transportation price f* (f* ≥ 0) is not satisfied in the monopolistic case.

## Numerical analysis under the quadratic cost function

In this section, we numerically confirm that the comparative statics derived in Section 4 are also valid under a general quadratic cost function. Under the general cost function, unlike the cost function specified in the previous sections, there are multiple equilibria for the optimal product R&D investment *d*_*i*_. Among the multiple equilibria, there is an equilibrium that is completely consistent with the comparative statics in Section 4. Additionally, this equilibrium becomes the unique equilibrium when the efficiency of product differentiation *e* is high.

We assume that the R&D cost function is quadratic and that *c*(*d*_*i*_, *e*) becomes as follows:

$$\begin{array}{c} c(d_{i},e)≔\frac{1}{e}\frac{d_{i}^{2}}{2}. \end{array}$$

Using this R&D cost function, we derive the optimal product R&D investment $d_{i}^{**}$ that satisfies Eq (11), i.e.,

$$\begin{array}{c} \frac{d_{i}^{**}}{e}=\frac{2a^{2}(11-d_{i}^{**})}{9{\left(3-d_{i}^{**}\right)}^{3}}. \end{array}$$

Under the assumption of parameter *a* = 1, the above equation becomes

$$\begin{array}{c} -9{d_{i}^{**}}^{4}+81{d_{i}^{**}}^{3}-243{d_{i}^{**}}^{2}+\left(2e+243\right)d_{i}^{**}-22e=0. \end{array}$$

Since this is a quartic equation, there are potentially four roots. Two of them are complex roots, and others are real roots, which we focus on. Unfortunately, both real roots are complicated, and it is difficult to present an explicit solution. Thus, we perform numerical analysis to determine the conditions under which given the efficiency of product differentiation *e*, each of them is well-defined, i.e., *d*_*i*_ ∈ (0, 1). We then focus on the real root that exists under high efficiency of product differentiation and show that it is consistent with the comparative statics analyzed in Section 4.

**Fig 1:**
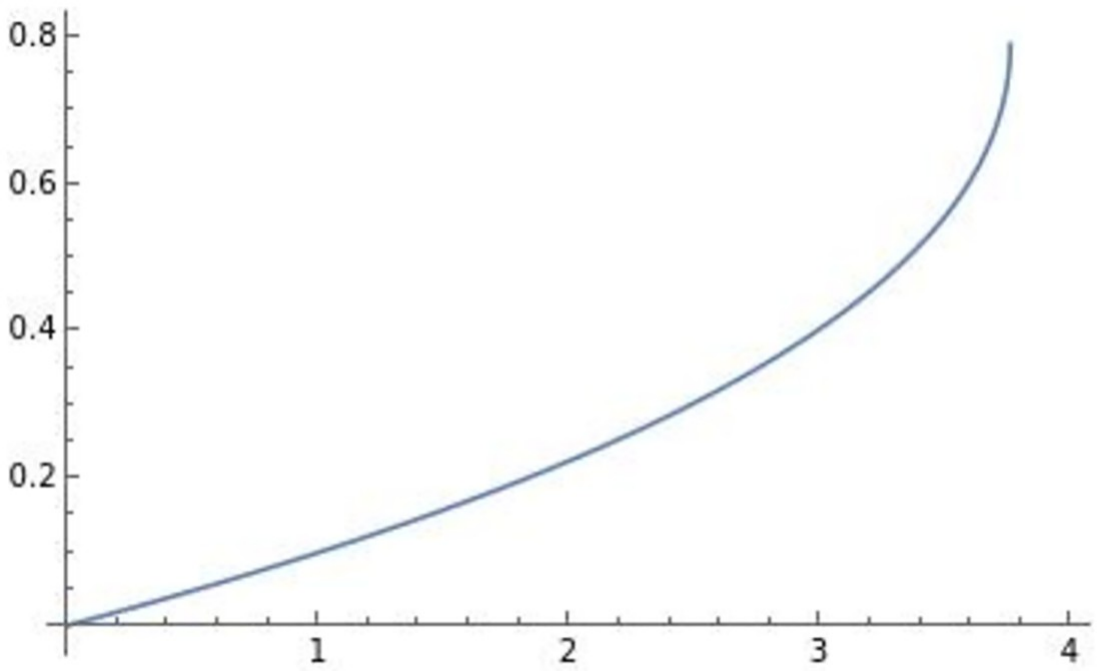
$\frac{\partial d_{i}^{**}}{\partial e}>0$ . The efficiency *e* positively affects the optimal product R&D investment $d_{i}^{**}$.

**Fig 2:**
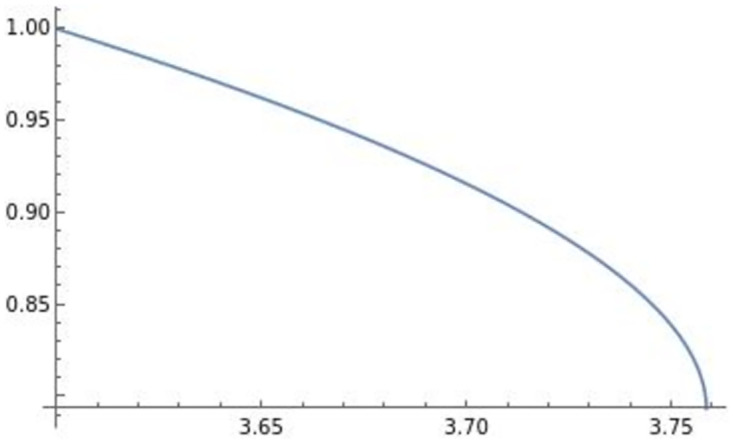
$\frac{\partial d_{i}^{**}}{\partial e}<0$ . The efficiency *e* negatively affects the optimal product R&D investment $d_{i}^{**}$.

Using Mathematica, we derive two different types of well-defined optimal $d_{i}^{**}$ drawn in Figs 1 and 2 as follows:

The horizontal axis in the figures indicates the efficiency of product differentiation *e*, the vertical axis indicates the level of product R&D investment $d_{i}^{**}$, and the blue lines in the figures illustrate $\left(e,d_{i}^{**}\right)$. In the following analysis, the horizontal axis is the efficiency of product differentiation *e*, the vertical axis represents the endogenous variables, and the blue line represents the effect of efficiency on an endogenous variable. The optimal product R&D investment $d_{i}^{**}$ in Fig 1 is the usual $d_{i}^{**}$, which is positively affected by efficiency *e*. On the other hand, similar to the result in Sections 3 and 4, the optimal product R&D investment $d_{i}^{**}$ in Fig 2, which we focus on, is negatively affected by efficiency *e*. This $d_{i}^{**}$ requires high efficiency of product differentiation *e* ($d_{i}^{**}$ is well-defined when *e* ∈ (3.6, 3.76) in Fig 2) relative to the usual level.

**Fig 3:**
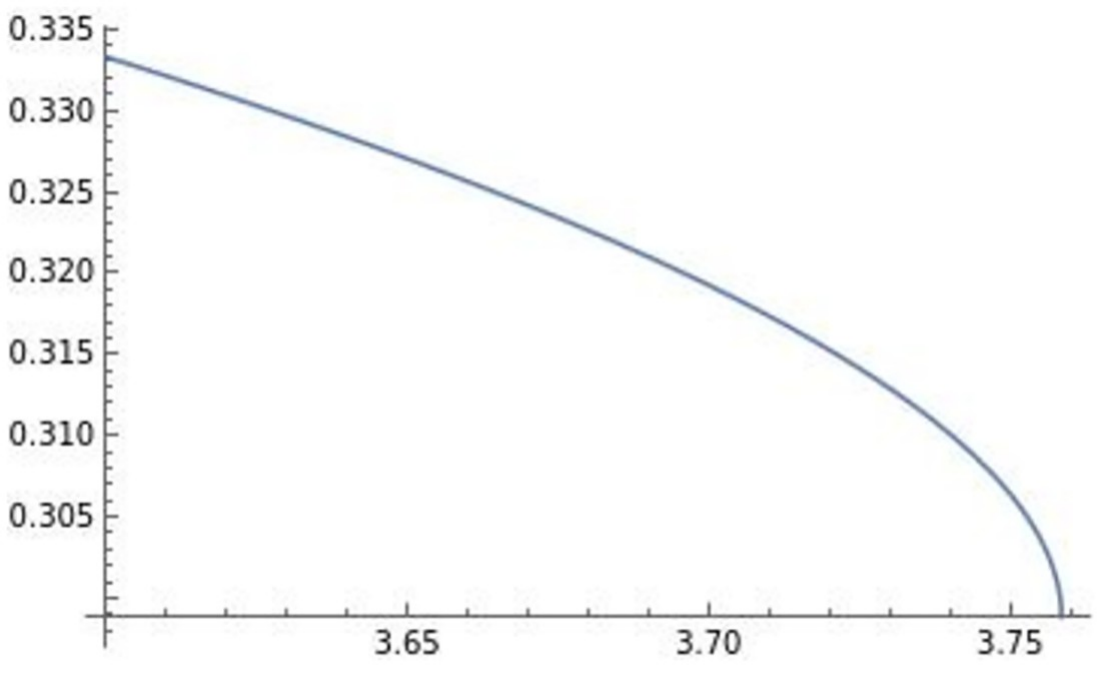
Transportation price. The optimal transportation price is negatively affected by efficiency *e*.

In the remainder of this section, focusing on the optimal $d_{i}^{**}$ in Fig 2, we numerically analyze the comparative statics of efficiency with respect to the optimal transportation price f**, the equilibrium profit of firm *i* $\pi_{i}^{**}$, and the level of trade $\frac{q_{ij}^{**}}{q_{ii}^{**}}$. The effect of efficiency *e* on the optimal transportation price f** is depicted in Fig 3.

**Fig 4:**
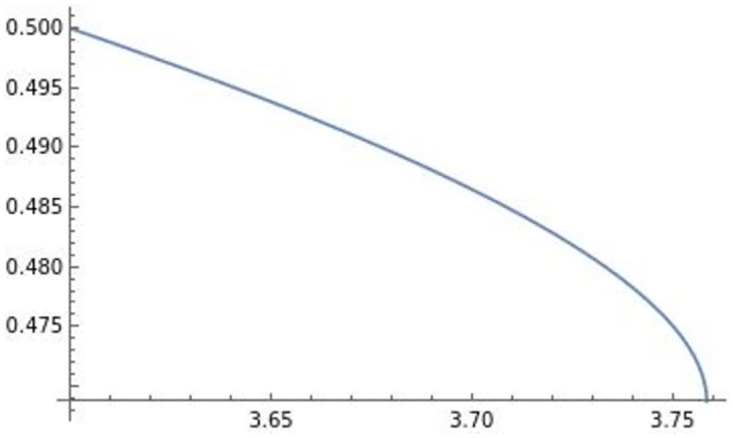
The optimal price of firm *i*’s product in country *i*: $p_{ii}^{**}$. The optimal price of firm *i*’s product in country *i* is negatively affected by efficiency *e*.

**Fig 5:**
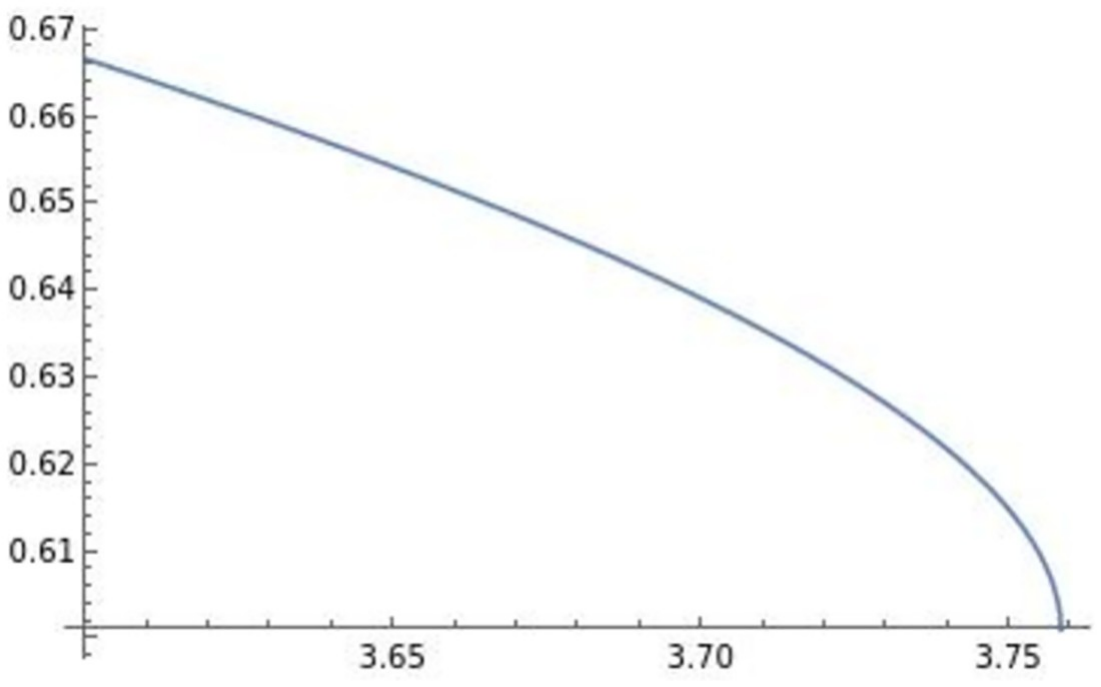
The optimal price of firm *i*’s product in country *j*: $p_{ij}^{**}$. The optimal price of firm *i*’s product in country *j* is negatively affected by efficiency *e*.

**Fig 6:**
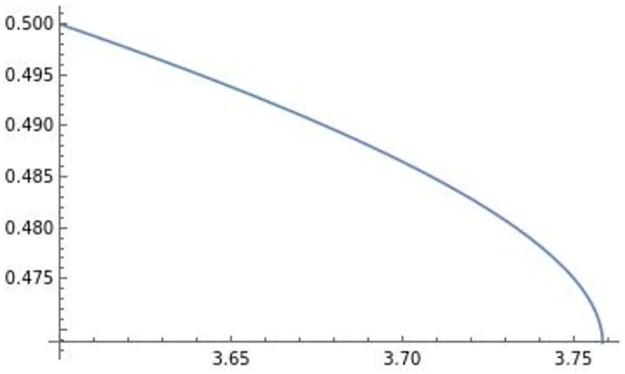
The optimal quantity of firm *i*’s product in country *i*: $q_{ii}^{**}$. The optimal quantity of firm *i*’s product in country *i* is negatively affected by efficiency *e*.

**Fig 7:**
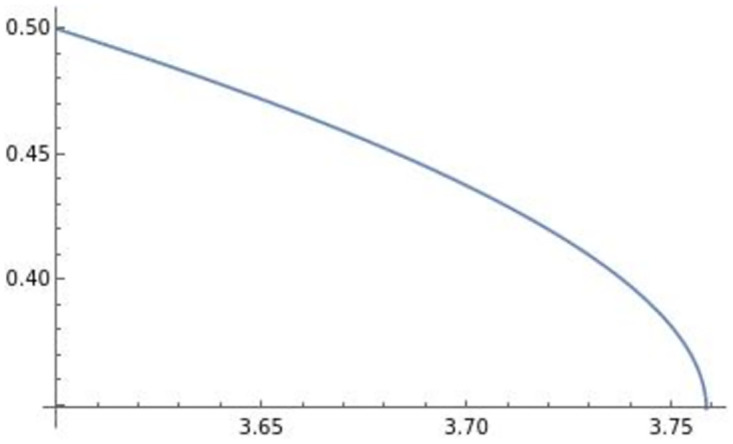
The optimal quantity of firm *i*’s product in country *j*: $q_{ij}^{**}$. The optimal quantity of firm *i*’s product in country *j* is negatively affected by efficiency *e*.

The optimal transportation price is negatively affected by efficiency *e*. Prior to deriving the profits and the level of trade, we depict the effect of efficiency *e* on $p_{ii}^{**},p_{ij}^{**},q_{ii}^{**},q_{ij}^{**}$ in Figs 4–7.

**Fig 8:**
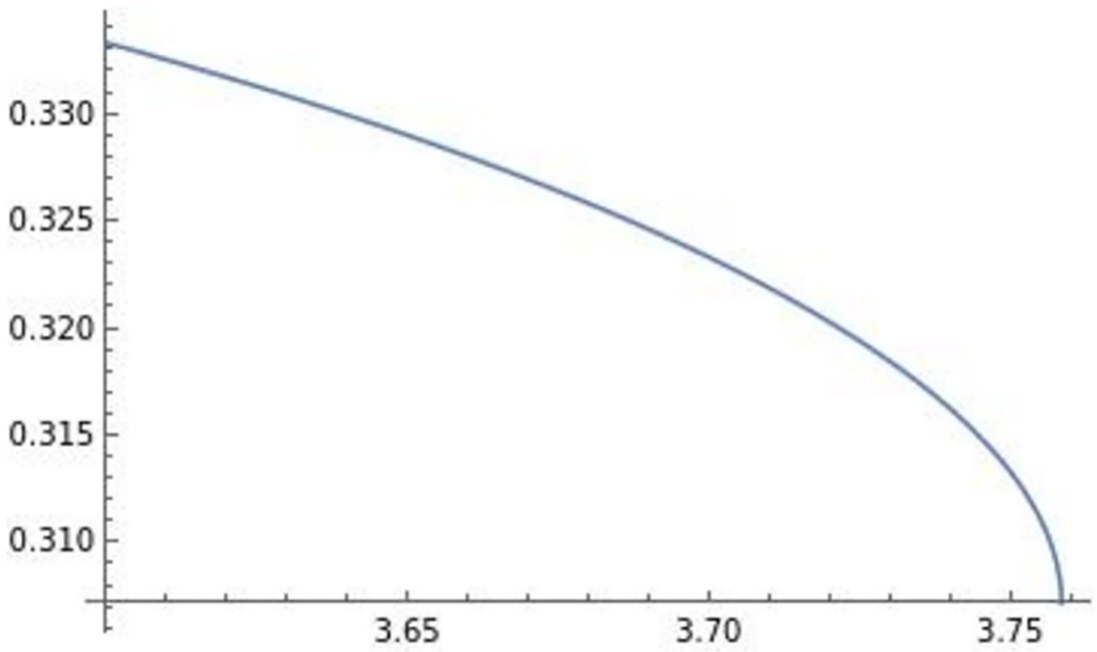
The optimal profit $\pi_{i}^{**}$. The optimal profit $\pi_{i}^{**}$ is negatively affected by efficiency *e*.

Using these, the effect of efficiency *e* on $\pi_{i}^{*}$ is depicted in Fig 8.

**Fig 9:**
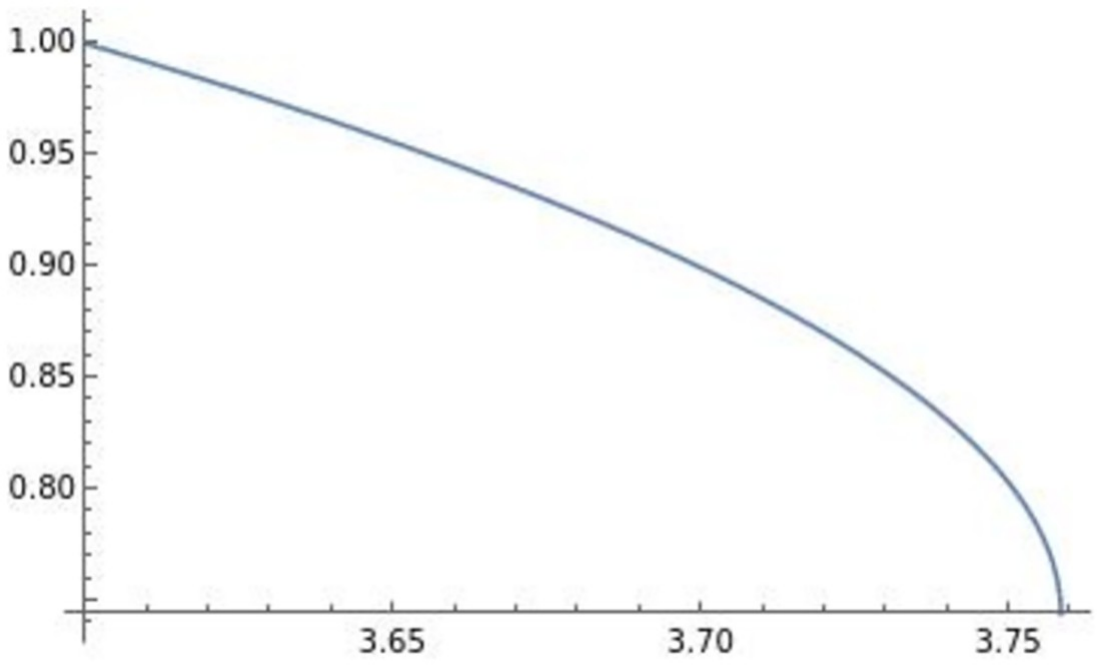
The level of trade $\frac{q_{ij}^{**}}{q_{ii}^{**}}$. The level of trade $\frac{q_{ij}^{**}}{q_{ii}^{**}}$ is negatively affected by efficiency *e*.

The effect of efficiency *e* on the level of trade $\frac{q_{ij}^{**}}{q_{ii}^{**}}$ is depicted in Fig 9.

In Fig 9, efficiency *e* negatively affects the level of trade $\frac{q_{ij}^{**}}{q_{ii}^{**}}$. Using Fig 2 and *s* = 1 − *d*_*i*_, this also implies that the inverse of the degree of differentiation *s* is positively associated with trade.

The above numerical comparative static results are fundamentally consistent with those of analytical comparative statistics under the specified cost function in Section 4.

## Conclusion

In this article, we constructed a symmetric international oligopoly model with endogenous product differentiation and endogenous transportation prices. We find that an increase in the efficiency of differentiation always decreases R&D investment and always decreases the level of differentiation. The impact of the decrease in the level of differentiation the domestic supply of firms always decreases. Furthermore, we find that a decrease in the level of differentiation always leads to a decrease in the amount of exports and a decrease in the transportation price. These results are similar to those of [12], who incorporates cost-reducing R&D. On the other hand, although the efficiency reduction of differentiation reduces both the total revenue and total cost of the firm, it always reduces the firm’s profit because the effect of the former exceeds the effect of the latter. This result is different from that of [12], who found that a firm’s profits decrease only when the efficiency *e* is relatively low.

This basic model could be extended to analyze an asymmetric case in which each country has identical R&D cost functions and identical (and positive) transportation prices. Since [2] indicated that transportation costs also declined in the second half of the twentieth century as a result of technological change, another extension would be to introduce cost-reducing R&D by the transporter. Additionally, comparing different types of competition (price and quantity) between firms and transporters could be an important extension of this model. This extension would provide the counterintuitive analytical and numerical comparative statistics derived in this article.

## Supporting information

S1 File (ZIP)

## References

[1] Krugman P. Scale economies, product differentiation, and the pattern of trade. American Economic Review 70.5 (1980): 950–959.

[2] Hummels D. Transportation costs and international trade in the second era of globalization. Journal of Economic Perspectives 21.3 (2007): 131–154. doi: 10.1257/jep.21.3.131

[3] Hummels D, Lugovskyy V, Skiba A. The trade reducing effects of market power in international shipping. Journal of Development Economics 89.1 (2009): 84–97. doi: 10.1016/j.jdeveco.2008.05.001

[4] Lin P, Saggi K. Product differentiation, process R&D, and the nature of market competition. European Economic Review 46.1 (2002): 201–211. doi: 10.1016/S0014-2921(00)00090-8

[5] Francois JF, Wooton I. Trade in international transport services: the role of competition. Review of International Economics 9.2 (2001): 249–261. doi: 10.1111/1467-9396.00277

[6] Andriamananjara S. Trade and International Transport Services: an Analytical Framework. Journal of Economic Integration (2004): 604–625.

[7] Behrens K, Picard PM. Transportation, freight rates, and economic geography. Journal of International Economics 85.2 (2011): 280–291. doi: 10.1016/j.jinteco.2011.06.003

[8] Takahashi T. Directional imbalance in transport prices and economic geography. Journal of Urban Economics 69.1 (2011): 92–102. doi: 10.1016/j.jue.2010.08.003

[9] Abe K, Hattori K, Kawagoshi Y. Trade liberalization and environmental regulation on international transportation. Japanese Economic Review 65.4 (2014): 468–482. doi: 10.1111/jere.12044

[10] Forslid R, Okubo T. Which firms are left in the periphery? Spatial sorting of heterogeneous firms with scale economies in transportation. Journal of Regional Science 55.1 (2015): 51–65. doi: 10.1111/jors.12115

[11] Ishikawa J, Tarui N. Backfiring with backhaul problems: Trade and industrial policies with endogenous transport costs. Journal of International Economics 111 (2018): 81–98. doi: 10.1016/j.jinteco.2017.12.004

[12] Takauchi K. Endogenous transport price and international R&D rivalry. Economic Modelling 46 (2015): 36–43. doi: 10.1016/j.econmod.2014.12.019

[13] Takauchi K, Mizuno T. Solving a hold-up problem may harm all firms: Downstream R&D and transport-price contracts. International Review of Economics and Finance,59 (2019): 29–49. doi: 10.1016/j.iref.2018.08.002

[14] Takauchi K, Mizuno, T. Consumer-benefiting transport cost: The role of product innovation in a vertical structure. No. 2017. Graduate School of Economics, Kobe University, 2020.

[15] Garcia-Macia D, Hsieh CT, Klenow PJ. How destructive is innovation?. Econometrica 87(5) (2019): 1507–1541. doi: 10.3982/ECTA14930

[16] Greenwood J, Hercowitz Z, Krusell P. Long-run implications of investment-specific technological change. American Economic Review 87.3 (1997): 342.

[17] Fujita M, Thisse J-F. Economics of Agglomeration 2nd edition. Cambridge University Press, 2013.

[18] Rosenkranz S. Simultaneous choice of process and product innovation when consumers have a preference for product variety. Journal of Economic Behavior and Organization 50.2 (2003): 183–201. doi: 10.1016/S0167-2681(02)00047-1

[19] Singh N, Vives X. Price and Quantity Competition in a Differentiated Duopoly. Rand Journal of Economics (1984): 546–554.

[20] Bastos P, Straume OR. Globalization, product differentiation, and wage inequality. Canadian Journal of Economics 45.3 (2012): 857–878. doi: 10.1111/j.1540-5982.2012.01718.x

[21] Brander JA, Spencer BJ. Intra-industry trade with Bertrand and Cournot oligopoly: The role of endogenous horizontal product differentiation. Research in Economics 69.2 (2015): 157–165. doi: 10.1016/j.rie.2015.02.007

