# Supplementary material for: Transportation price, product differentiation, and R&D in an oligopoly
Source: PLoS One. 2022 Sep 9;17(9):e0273904. doi: 10.1371/journal.pone.0273904 (PMC9462559; doi:10.1371/journal.pone.0273904)

In[1]:=

```
(*setup for deriving the solution*)
Solve[-1/e + (2*a^2*(11 - d))/(9*(3 - d)^3*d) == 0, d]
```

Out[1]=

$$\left\{\left\{d\to\frac{9}{4}-\frac{1}{2}\sqrt{\left(\frac{9}{4}+\frac{35\times 2^{2/3}\,a^2\,e}{3\times 3^{1/3}\left(972\,a^2\,e+3\,a^4\,e^2-\sqrt{3}\,\sqrt{314\,928\,a^4\,e^2-83\,806\,a^6\,e^3+3\,a^8\,e^4}\right)^{1/3}}+\frac{2^{1/3}\left(972\,a^2\,e+3\,a^4\,e^2-\sqrt{3}\,\sqrt{314\,928\,a^4\,e^2-83\,806\,a^6\,e^3+3\,a^8\,e^4}\right)^{1/3}}{3\times 3^{2/3}}\right)}-\right.\right.$$

$$\frac{1}{2}\sqrt{\left(\frac{9}{2}-\frac{35\times 2^{2/3}\,a^2\,e}{3\times 3^{1/3}\left(972\,a^2\,e+3\,a^4\,e^2-\sqrt{3}\,\sqrt{314\,928\,a^4\,e^2-83\,806\,a^6\,e^3+3\,a^8\,e^4}\right)^{1/3}}-\frac{2^{1/3}\left(972\,a^2\,e+3\,a^4\,e^2-\sqrt{3}\,\sqrt{314\,928\,a^4\,e^2-83\,806\,a^6\,e^3+3\,a^8\,e^4}\right)^{1/3}}{3\times 3^{2/3}}-\left(-243-\frac{8}{9}\left(-243-2\,a^2\,e\right)\right)\Big/\left(4\sqrt{\left(\frac{9}{4}+\frac{35\times 2^{2/3}\,a^2\,e}{3\times 3^{1/3}\left(972\,a^2\,e+3\,a^4\,e^2-\sqrt{3}\,\sqrt{314\,928\,a^4\,e^2-83\,806\,a^6\,e^3+3\,a^8\,e^4}\right)^{1/3}}+\frac{2^{1/3}\left(972\,a^2\,e+3\,a^4\,e^2-\sqrt{3}\,\sqrt{314\,928\,a^4\,e^2-83\,806\,a^6\,e^3+3\,a^8\,e^4}\right)^{1/3}}{3\times 3^{2/3}}\right)}\right)\right)}\right\},$$

$$\left\{d\to\frac{9}{4}-\frac{1}{2}\sqrt{\left(\frac{9}{4}+\frac{35\times 2^{2/3}\,a^2\,e}{3\times 3^{1/3}\left(972\,a^2\,e+3\,a^4\,e^2-\sqrt{3}\,\sqrt{314\,928\,a^4\,e^2-83\,806\,a^6\,e^3+3\,a^8\,e^4}\right)^{1/3}}+\frac{2^{1/3}\left(972\,a^2\,e+3\,a^4\,e^2-\sqrt{3}\,\sqrt{314\,928\,a^4\,e^2-83\,806\,a^6\,e^3+3\,a^8\,e^4}\right)^{1/3}}{3\times 3^{2/3}}\right)}+\right.$$

$$\frac{1}{2}\sqrt{\left(\frac{9}{2}-\frac{35\times 2^{2/3}\,a^2\,e}{3\times 3^{1/3}\left(972\,a^2\,e+3\,a^4\,e^2-\sqrt{3}\,\sqrt{314\,928\,a^4\,e^2-83\,806\,a^6\,e^3+3\,a^8\,e^4}\right)^{1/3}}-\right.}$$

$$\frac{2^{1/3}\left(972\ a^2\ e+3\ a^4\ e^2-\sqrt{3}\ \sqrt{314\,928\ a^4\ e^2-83\,806\ a^6\ e^3+3\ a^8\ e^4}\right)^{1/3}}{3\times 3^{2/3}}-$$

$$\left(-243-\frac{8}{9}\left(-243-2\ a^2\ e\right)\right)\Big/$$

$$\left(4\sqrt{\left(\frac{9}{4}+\frac{35\times 2^{2/3}\ a^2\ e}{3\times 3^{1/3}\left(972\ a^2\ e+3\ a^4\ e^2-\sqrt{3}\ \sqrt{314\,928\ a^4\ e^2-83\,806\ a^6\ e^3+3\ a^8\ e^4}\right)^{1/3}}+\right.}\right.$$

$$\left.\left.\left.\left.\frac{2^{1/3}\left(972\ a^2\ e+3\ a^4\ e^2-\sqrt{3}\ \sqrt{314\,928\ a^4\ e^2-83\,806\ a^6\ e^3+3\ a^8\ e^4}\right)^{1/3}}{3\times 3^{2/3}}\right)\right)\right)\right\},$$

$$\left\{d\to\frac{9}{4}+\frac{1}{2}\sqrt{\left(\frac{9}{4}+\frac{35\times 2^{2/3}\ a^2\ e}{3\times 3^{1/3}\left(972\ a^2\ e+3\ a^4\ e^2-\sqrt{3}\ \sqrt{314\,928\ a^4\ e^2-83\,806\ a^6\ e^3+3\ a^8\ e^4}\right)^{1/3}}+\right.}\right.$$

$$\left.\frac{2^{1/3}\left(972\ a^2\ e+3\ a^4\ e^2-\sqrt{3}\ \sqrt{314\,928\ a^4\ e^2-83\,806\ a^6\ e^3+3\ a^8\ e^4}\right)^{1/3}}{3\times 3^{2/3}}\right)-$$

$$\frac{1}{2}\sqrt{\left(\frac{9}{2}-\frac{35\times 2^{2/3}\ a^2\ e}{3\times 3^{1/3}\left(972\ a^2\ e+3\ a^4\ e^2-\sqrt{3}\ \sqrt{314\,928\ a^4\ e^2-83\,806\ a^6\ e^3+3\ a^8\ e^4}\right)^{1/3}}-\right.}$$

$$\frac{2^{1/3}\left(972\ a^2\ e+3\ a^4\ e^2-\sqrt{3}\ \sqrt{314\,928\ a^4\ e^2-83\,806\ a^6\ e^3+3\ a^8\ e^4}\right)^{1/3}}{3\times 3^{2/3}}+$$

$$\left(-243-\frac{8}{9}\left(-243-2\ a^2\ e\right)\right)\Big/$$

$$\left(4\sqrt{\left(\frac{9}{4}+\frac{35\times 2^{2/3}\ a^2\ e}{3\times 3^{1/3}\left(972\ a^2\ e+3\ a^4\ e^2-\sqrt{3}\ \sqrt{314\,928\ a^4\ e^2-83\,806\ a^6\ e^3+3\ a^8\ e^4}\right)^{1/3}}+\right.}\right.$$

$$\left.\left.\left.\left.\frac{2^{1/3}\left(972\ a^2\ e+3\ a^4\ e^2-\sqrt{3}\ \sqrt{314\,928\ a^4\ e^2-83\,806\ a^6\ e^3+3\ a^8\ e^4}\right)^{1/3}}{3\times 3^{2/3}}\right)\right)\right)\right\},$$

$$\left\{d\to\frac{9}{4}+\frac{1}{2}\sqrt{\left(\frac{9}{4}+\frac{35\times 2^{2/3}\ a^2\ e}{3\times 3^{1/3}\left(972\ a^2\ e+3\ a^4\ e^2-\sqrt{3}\ \sqrt{314\,928\ a^4\ e^2-83\,806\ a^6\ e^3+3\ a^8\ e^4}\right)^{1/3}}+\right.}\right.$$

$$\left.\frac{2^{1/3}\left(972\ a^2\ e+3\ a^4\ e^2-\sqrt{3}\ \sqrt{314\,928\ a^4\ e^2-83\,806\ a^6\ e^3+3\ a^8\ e^4}\right)^{1/3}}{3\times 3^{2/3}}\right)+$$

$$\frac{1}{2}\sqrt{\left(\frac{9}{2}-\frac{35\times 2^{2/3}\ a^2\ e}{3\times 3^{1/3}\left(972\ a^2\ e+3\ a^4\ e^2-\sqrt{3}\ \sqrt{314\,928\ a^4\ e^2-83\,806\ a^6\ e^3+3\ a^8\ e^4}\right)^{1/3}}-\right.}$$

$$\frac{2^{1/3}\left(972\ a^2\ e+3\ a^4\ e^2-\sqrt{3}\ \sqrt{314\,928\ a^4\ e^2-83\,806\ a^6\ e^3+3\ a^8\ e^4}\right)^{1/3}}{3\times 3^{2/3}}+$$

$$\left(-243-\frac{8}{9}\left(-243-2\ a^2\ e\right)\right)\Bigg/$$

$$\left(4\sqrt{\left(\frac{9}{4}+\frac{35\times 2^{2/3}\ a^2\ e}{3\times 3^{1/3}\left(972\ a^2\ e+3\ a^4\ e^2-\sqrt{3}\ \sqrt{314\,928\ a^4\ e^2-83\,806\ a^6\ e^3+3\ a^8\ e^4}\right)^{1/3}}+\right.}\right.$$

$$\left.\left.\left.\left.\frac{2^{1/3}\left(972\ a^2\ e+3\ a^4\ e^2-\sqrt{3}\ \sqrt{314\,928\ a^4\ e^2-83\,806\ a^6\ e^3+3\ a^8\ e^4}\right)^{1/3}}{3\times 3^{2/3}}\right)\right)\right)\right\}\Big\}$$

In[2]:=

$$d = \frac{9}{4} - \frac{1}{2}\sqrt{\left(\frac{9}{4} + \frac{35 \times 2^{2/3} a^2 e}{3 \times 3^{1/3}\left(972\,a^2 e + 3\,a^4 e^2 - \sqrt{3}\,\sqrt{314\,928\,a^4 e^2 - 83\,806\,a^6 e^3 + 3\,a^8 e^4}\right)^{1/3}} + \frac{2^{1/3}\left(972\,a^2 e + 3\,a^4 e^2 - \sqrt{3}\,\sqrt{314\,928\,a^4 e^2 - 83\,806\,a^6 e^3 + 3\,a^8 e^4}\right)^{1/3}}{3 \times 3^{2/3}}\right)} - \frac{1}{2}\sqrt{\left(\frac{9}{2} - \frac{35 \times 2^{2/3} a^2 e}{3 \times 3^{1/3}\left(972\,a^2 e + 3\,a^4 e^2 - \sqrt{3}\,\sqrt{314\,928\,a^4 e^2 - 83\,806\,a^6 e^3 + 3\,a^8 e^4}\right)^{1/3}} - \frac{2^{1/3}\left(972\,a^2 e + 3\,a^4 e^2 - \sqrt{3}\,\sqrt{314\,928\,a^4 e^2 - 83\,806\,a^6 e^3 + 3\,a^8 e^4}\right)^{1/3}}{3 \times 3^{2/3}} - \left(-243 - \frac{8}{9}\left(-243 - 2\,a^2 e\right)\right) \Bigg/ \left(4\sqrt{\left(\frac{9}{4} + \frac{35 \times 2^{2/3} a^2 e}{3 \times 3^{1/3}\left(972\,a^2 e + 3\,a^4 e^2 - \sqrt{3}\,\sqrt{314\,928\,a^4 e^2 - 83\,806\,a^6 e^3 + 3\,a^8 e^4}\right)^{1/3}} + \frac{2^{1/3}\left(972\,a^2 e + 3\,a^4 e^2 - \sqrt{3}\,\sqrt{314\,928\,a^4 e^2 - 83\,806\,a^6 e^3 + 3\,a^8 e^4}\right)^{1/3}}{3 \times 3^{2/3}}\right)}\right)\right)}$$

Out[2]=

$$\frac{9}{4}-\frac{1}{2}\sqrt{\left(\frac{9}{4}+\frac{35\times 2^{2/3}\,a^2\,e}{3\times 3^{1/3}\left(972\,a^2\,e+3\,a^4\,e^2-\sqrt{3}\,\sqrt{314\,928\,a^4\,e^2-83\,806\,a^6\,e^3+3\,a^8\,e^4}\right)^{1/3}}+\frac{2^{1/3}\left(972\,a^2\,e+3\,a^4\,e^2-\sqrt{3}\,\sqrt{314\,928\,a^4\,e^2-83\,806\,a^6\,e^3+3\,a^8\,e^4}\right)^{1/3}}{3\times 3^{2/3}}\right)}-$$

$$\frac{1}{2}\sqrt{\left(\frac{9}{2}-\frac{35\times 2^{2/3}\,a^2\,e}{3\times 3^{1/3}\left(972\,a^2\,e+3\,a^4\,e^2-\sqrt{3}\,\sqrt{314\,928\,a^4\,e^2-83\,806\,a^6\,e^3+3\,a^8\,e^4}\right)^{1/3}}-\frac{2^{1/3}\left(972\,a^2\,e+3\,a^4\,e^2-\sqrt{3}\,\sqrt{314\,928\,a^4\,e^2-83\,806\,a^6\,e^3+3\,a^8\,e^4}\right)^{1/3}}{3\times 3^{2/3}}-\left(-243-\frac{8}{9}\left(-243-2\,a^2\,e\right)\right)\Big/\left(4\sqrt{\left(\frac{9}{4}+\frac{35\times 2^{2/3}\,a^2\,e}{3\times 3^{1/3}\left(972\,a^2\,e+3\,a^4\,e^2-\sqrt{3}\,\sqrt{314\,928\,a^4\,e^2-83\,806\,a^6\,e^3+3\,a^8\,e^4}\right)^{1/3}}+\frac{2^{1/3}\left(972\,a^2\,e+3\,a^4\,e^2-\sqrt{3}\,\sqrt{314\,928\,a^4\,e^2-83\,806\,a^6\,e^3+3\,a^8\,e^4}\right)^{1/3}}{3\times 3^{2/3}}\right)}\right)\right)}$$

In[3]:=
```
(*When we assume a=1, first solution becomes*)
d /. {a → 1}
```

Out[3]=

$$\frac{9}{4}-\frac{1}{2}\sqrt{\left(\frac{9}{4}+\frac{35\times 2^{2/3}\,e}{3\times 3^{1/3}\left(972\,e+3\,e^2-\sqrt{3}\,\sqrt{314928\,e^2-83806\,e^3+3\,e^4}\right)^{1/3}}+\frac{2^{1/3}\left(972\,e+3\,e^2-\sqrt{3}\,\sqrt{314928\,e^2-83806\,e^3+3\,e^4}\right)^{1/3}}{3\times 3^{2/3}}\right)}-\frac{1}{2}\sqrt{\left(\frac{9}{2}-\frac{35\times 2^{2/3}\,e}{3\times 3^{1/3}\left(972\,e+3\,e^2-\sqrt{3}\,\sqrt{314928\,e^2-83806\,e^3+3\,e^4}\right)^{1/3}}-\frac{2^{1/3}\left(972\,e+3\,e^2-\sqrt{3}\,\sqrt{314928\,e^2-83806\,e^3+3\,e^4}\right)^{1/3}}{3\times 3^{2/3}}-\frac{-243-\frac{8}{9}\left(-243-2\,e\right)}{4\sqrt{\frac{9}{4}+\frac{35\times 2^{2/3}\,e}{3\times 3^{1/3}\left(972\,e+3\,e^2-\sqrt{3}\,\sqrt{314928\,e^2-83806\,e^3+3\,e^4}\right)^{1/3}}+\frac{2^{1/3}\left(972\,e+3\,e^2-\sqrt{3}\,\sqrt{314928\,e^2-83806\,e^3+3\,e^4}\right)^{1/3}}{3\times 3^{2/3}}}}\right)}$$

In[4]:=
```
(*Plot this from e=0 to e=4*)
Plot[{%3}, {e, 0, 4}]
```

Out[4]=

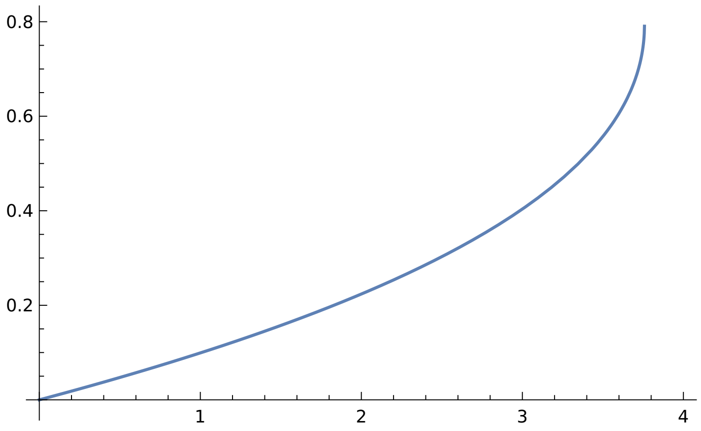

In[1]:= $\text{Solve}\left[-1/e + \frac{2 * a^2 * (11 - d)}{9 * (3 - d)^3 * d} == 0, d\right]$

Out[1]=

$$\Bigg\{\Bigg\{d \to \frac{9}{4} - \frac{1}{2}\sqrt{\left(\frac{9}{4} + \frac{35 \times 2^{2/3}\, a^2\, e}{3 \times 3^{1/3}\left(972\, a^2\, e + 3\, a^4\, e^2 - \sqrt{3}\ \sqrt{314\,928\, a^4\, e^2 - 83\,806\, a^6\, e^3 + 3\, a^8\, e^4}\right)^{1/3}} + \frac{2^{1/3}\left(972\, a^2\, e + 3\, a^4\, e^2 - \sqrt{3}\ \sqrt{314\,928\, a^4\, e^2 - 83\,806\, a^6\, e^3 + 3\, a^8\, e^4}\right)^{1/3}}{3 \times 3^{2/3}}\right)} -$$

$$\frac{1}{2}\sqrt{\left(\frac{9}{2} - \frac{35 \times 2^{2/3}\, a^2\, e}{3 \times 3^{1/3}\left(972\, a^2\, e + 3\, a^4\, e^2 - \sqrt{3}\ \sqrt{314\,928\, a^4\, e^2 - 83\,806\, a^6\, e^3 + 3\, a^8\, e^4}\right)^{1/3}} - \frac{2^{1/3}\left(972\, a^2\, e + 3\, a^4\, e^2 - \sqrt{3}\ \sqrt{314\,928\, a^4\, e^2 - 83\,806\, a^6\, e^3 + 3\, a^8\, e^4}\right)^{1/3}}{3 \times 3^{2/3}} - \right.}$$

$$\left(-243 - \frac{8}{9}\left(-243 - 2\, a^2\, e\right)\right) \Bigg/$$

$$\left.\left(4\sqrt{\left(\frac{9}{4} + \frac{35 \times 2^{2/3}\, a^2\, e}{3 \times 3^{1/3}\left(972\, a^2\, e + 3\, a^4\, e^2 - \sqrt{3}\ \sqrt{314\,928\, a^4\, e^2 - 83\,806\, a^6\, e^3 + 3\, a^8\, e^4}\right)^{1/3}} + \frac{2^{1/3}\left(972\, a^2\, e + 3\, a^4\, e^2 - \sqrt{3}\ \sqrt{314\,928\, a^4\, e^2 - 83\,806\, a^6\, e^3 + 3\, a^8\, e^4}\right)^{1/3}}{3 \times 3^{2/3}}\right)}\right)\right)\Bigg\},$$

$$\Bigg\{d \to \frac{9}{4} - \frac{1}{2}\sqrt{\left(\frac{9}{4} + \frac{35 \times 2^{2/3}\, a^2\, e}{3 \times 3^{1/3}\left(972\, a^2\, e + 3\, a^4\, e^2 - \sqrt{3}\ \sqrt{314\,928\, a^4\, e^2 - 83\,806\, a^6\, e^3 + 3\, a^8\, e^4}\right)^{1/3}} + \frac{2^{1/3}\left(972\, a^2\, e + 3\, a^4\, e^2 - \sqrt{3}\ \sqrt{314\,928\, a^4\, e^2 - 83\,806\, a^6\, e^3 + 3\, a^8\, e^4}\right)^{1/3}}{3 \times 3^{2/3}}\right)} +$$

$$\frac{1}{2}\sqrt{\left(\frac{9}{2} - \frac{35 \times 2^{2/3}\, a^2\, e}{3 \times 3^{1/3}\left(972\, a^2\, e + 3\, a^4\, e^2 - \sqrt{3}\ \sqrt{314\,928\, a^4\, e^2 - 83\,806\, a^6\, e^3 + 3\, a^8\, e^4}\right)^{1/3}} - \frac{2^{1/3}\left(972\, a^2\, e + 3\, a^4\, e^2 - \sqrt{3}\ \sqrt{314\,928\, a^4\, e^2 - 83\,806\, a^6\, e^3 + 3\, a^8\, e^4}\right)^{1/3}}{3 \times 3^{2/3}} - \right.}$$

$$\left(-243-\frac{8}{9}\left(-243-2\,a^2\,e\right)\right)\Big/$$

$$\Bigg(4\sqrt{\Bigg(\frac{9}{4}+\frac{35\times 2^{2/3}\,a^2\,e}{3\times 3^{1/3}\left(972\,a^2\,e+3\,a^4\,e^2-\sqrt{3}\,\sqrt{314\,928\,a^4\,e^2-83\,806\,a^6\,e^3+3\,a^8\,e^4}\right)^{1/3}}+}$$

$$\frac{2^{1/3}\left(972\,a^2\,e+3\,a^4\,e^2-\sqrt{3}\,\sqrt{314\,928\,a^4\,e^2-83\,806\,a^6\,e^3+3\,a^8\,e^4}\right)^{1/3}}{3\times 3^{2/3}}\Bigg)\Bigg)\Bigg)\Big\},$$

$$\Big\{d\to\frac{9}{4}+\frac{1}{2}\sqrt{\Bigg(\frac{9}{4}+\frac{35\times 2^{2/3}\,a^2\,e}{3\times 3^{1/3}\left(972\,a^2\,e+3\,a^4\,e^2-\sqrt{3}\,\sqrt{314\,928\,a^4\,e^2-83\,806\,a^6\,e^3+3\,a^8\,e^4}\right)^{1/3}}+}$$

$$\frac{2^{1/3}\left(972\,a^2\,e+3\,a^4\,e^2-\sqrt{3}\,\sqrt{314\,928\,a^4\,e^2-83\,806\,a^6\,e^3+3\,a^8\,e^4}\right)^{1/3}}{3\times 3^{2/3}}\Bigg)-$$

$$\frac{1}{2}\sqrt{\Bigg(\frac{9}{2}-\frac{35\times 2^{2/3}\,a^2\,e}{3\times 3^{1/3}\left(972\,a^2\,e+3\,a^4\,e^2-\sqrt{3}\,\sqrt{314\,928\,a^4\,e^2-83\,806\,a^6\,e^3+3\,a^8\,e^4}\right)^{1/3}}-}$$

$$\frac{2^{1/3}\left(972\,a^2\,e+3\,a^4\,e^2-\sqrt{3}\,\sqrt{314\,928\,a^4\,e^2-83\,806\,a^6\,e^3+3\,a^8\,e^4}\right)^{1/3}}{3\times 3^{2/3}}+$$

$$\left(-243-\frac{8}{9}\left(-243-2\,a^2\,e\right)\right)\Big/$$

$$\Bigg(4\sqrt{\Bigg(\frac{9}{4}+\frac{35\times 2^{2/3}\,a^2\,e}{3\times 3^{1/3}\left(972\,a^2\,e+3\,a^4\,e^2-\sqrt{3}\,\sqrt{314\,928\,a^4\,e^2-83\,806\,a^6\,e^3+3\,a^8\,e^4}\right)^{1/3}}+}$$

$$\frac{2^{1/3}\left(972\,a^2\,e+3\,a^4\,e^2-\sqrt{3}\,\sqrt{314\,928\,a^4\,e^2-83\,806\,a^6\,e^3+3\,a^8\,e^4}\right)^{1/3}}{3\times 3^{2/3}}\Bigg)\Bigg)\Bigg)\Big\},$$

$$\Big\{d\to\frac{9}{4}+\frac{1}{2}\sqrt{\Bigg(\frac{9}{4}+\frac{35\times 2^{2/3}\,a^2\,e}{3\times 3^{1/3}\left(972\,a^2\,e+3\,a^4\,e^2-\sqrt{3}\,\sqrt{314\,928\,a^4\,e^2-83\,806\,a^6\,e^3+3\,a^8\,e^4}\right)^{1/3}}+}$$

$$\frac{2^{1/3}\left(972\,a^2\,e+3\,a^4\,e^2-\sqrt{3}\,\sqrt{314\,928\,a^4\,e^2-83\,806\,a^6\,e^3+3\,a^8\,e^4}\right)^{1/3}}{3\times 3^{2/3}}\Bigg)+$$

$$\frac{1}{2}\sqrt{\left(\frac{9}{2}-\frac{35\times 2^{2/3}\,a^2\,e}{3\times 3^{1/3}\left(972\,a^2\,e+3\,a^4\,e^2-\sqrt{3}\,\sqrt{314\,928\,a^4\,e^2-83\,806\,a^6\,e^3+3\,a^8\,e^4}\right)^{1/3}}-\right.}$$

$$\frac{2^{1/3}\left(972\,a^2\,e+3\,a^4\,e^2-\sqrt{3}\,\sqrt{314\,928\,a^4\,e^2-83\,806\,a^6\,e^3+3\,a^8\,e^4}\right)^{1/3}}{3\times 3^{2/3}}+$$

$$\left(-243-\frac{8}{9}\left(-243-2\,a^2\,e\right)\right)\Big/$$

$$\left(4\sqrt{\left(\frac{9}{4}+\frac{35\times 2^{2/3}\,a^2\,e}{3\times 3^{1/3}\left(972\,a^2\,e+3\,a^4\,e^2-\sqrt{3}\,\sqrt{314\,928\,a^4\,e^2-83\,806\,a^6\,e^3+3\,a^8\,e^4}\right)^{1/3}}+\right.}\right.$$

$$\left.\left.\left.\frac{2^{1/3}\left(972\,a^2\,e+3\,a^4\,e^2-\sqrt{3}\,\sqrt{314\,928\,a^4\,e^2-83\,806\,a^6\,e^3+3\,a^8\,e^4}\right)^{1/3}}{3\times 3^{2/3}}\right)\right)\right)\}\}$$

In[8]:= (*Second solution becomes*)

$$d = \frac{9}{4} - \frac{1}{2}\sqrt{\left(\frac{9}{4} + \frac{35 \times 2^{2/3} a^2 e}{3 \times 3^{1/3} \left(972 a^2 e + 3 a^4 e^2 - \sqrt{3}\ \sqrt{314\,928 a^4 e^2 - 83\,806 a^6 e^3 + 3 a^8 e^4}\right)^{1/3}} + \frac{2^{1/3} \left(972 a^2 e + 3 a^4 e^2 - \sqrt{3}\ \sqrt{314\,928 a^4 e^2 - 83\,806 a^6 e^3 + 3 a^8 e^4}\right)^{1/3}}{3 \times 3^{2/3}}\right)} + \frac{1}{2}\sqrt{\left(\frac{9}{2} - \frac{35 \times 2^{2/3} a^2 e}{3 \times 3^{1/3} \left(972 a^2 e + 3 a^4 e^2 - \sqrt{3}\ \sqrt{314\,928 a^4 e^2 - 83\,806 a^6 e^3 + 3 a^8 e^4}\right)^{1/3}} - \frac{2^{1/3} \left(972 a^2 e + 3 a^4 e^2 - \sqrt{3}\ \sqrt{314\,928 a^4 e^2 - 83\,806 a^6 e^3 + 3 a^8 e^4}\right)^{1/3}}{3 \times 3^{2/3}} - \left(-243 - \frac{8}{9}\left(-243 - 2 a^2 e\right)\right) \Bigg/ \left(4\sqrt{\left(\frac{9}{4} + \frac{35 \times 2^{2/3} a^2 e}{3 \times 3^{1/3} \left(972 a^2 e + 3 a^4 e^2 - \sqrt{3}\ \sqrt{314\,928 a^4 e^2 - 83\,806 a^6 e^3 + 3 a^8 e^4}\right)^{1/3}} + \frac{2^{1/3} \left(972 a^2 e + 3 a^4 e^2 - \sqrt{3}\ \sqrt{314\,928 a^4 e^2 - 83\,806 a^6 e^3 + 3 a^8 e^4}\right)^{1/3}}{3 \times 3^{2/3}}\right)}\right)\right)} \; /.\ \{a \to 1\}$$

Out[8]=

$$\frac{9}{4}-\frac{1}{2}\sqrt{\left(\frac{9}{4}+\frac{35\times 2^{2/3}\,e}{3\times 3^{1/3}\left(972\,e+3\,e^2-\sqrt{3}\,\sqrt{314\,928\,e^2-83\,806\,e^3+3\,e^4}\right)^{1/3}}+\frac{2^{1/3}\left(972\,e+3\,e^2-\sqrt{3}\,\sqrt{314\,928\,e^2-83\,806\,e^3+3\,e^4}\right)^{1/3}}{3\times 3^{2/3}}\right)}+$$

$$\frac{1}{2}\sqrt{\left(\frac{9}{2}-\frac{35\times 2^{2/3}\,e}{3\times 3^{1/3}\left(972\,e+3\,e^2-\sqrt{3}\,\sqrt{314\,928\,e^2-83\,806\,e^3+3\,e^4}\right)^{1/3}}-\frac{2^{1/3}\left(972\,e+3\,e^2-\sqrt{3}\,\sqrt{314\,928\,e^2-83\,806\,e^3+3\,e^4}\right)^{1/3}}{3\times 3^{2/3}}-\frac{-243-\frac{8}{9}\left(-243-2\,e\right)}{4\sqrt{\frac{9}{4}+\frac{35\times 2^{2/3}\,e}{3\times 3^{1/3}\left(972\,e+3\,e^2-\sqrt{3}\,\sqrt{314\,928\,e^2-83\,806\,e^3+3\,e^4}\right)^{1/3}}+\frac{2^{1/3}\left(972\,e+3\,e^2-\sqrt{3}\,\sqrt{314\,928\,e^2-83\,806\,e^3+3\,e^4}\right)^{1/3}}{3\times 3^{2/3}}}}\right)}$$

In[12]:=

```
(*Plot this from e=3.6 to e=3.76*)
Plot[d, {e, 3.6, 3.76}]
```

Out[12]=

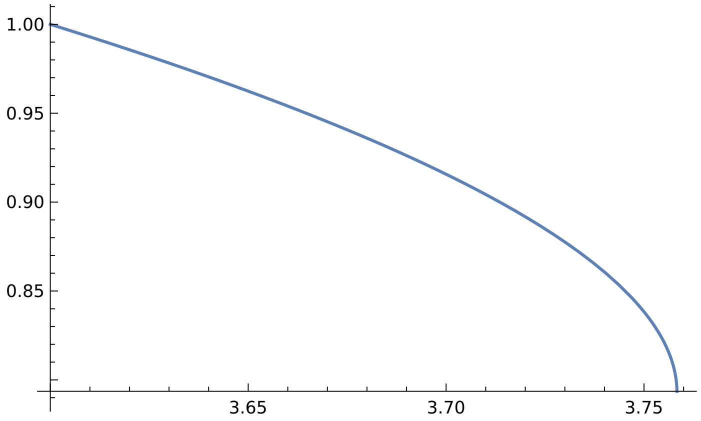

In[13]:=

```
(*setup for comparative statistics*)
s = 1 - d
```

Out[13]=

$$-\frac{5}{4}+\frac{1}{2}\sqrt{\left(\frac{9}{4}+\frac{35\times 2^{2/3}\,e}{3\times 3^{1/3}\left(972\,e+3\,e^2-\sqrt{3}\,\sqrt{314\,928\,e^2-83\,806\,e^3+3\,e^4}\right)^{1/3}}+\frac{2^{1/3}\left(972\,e+3\,e^2-\sqrt{3}\,\sqrt{314\,928\,e^2-83\,806\,e^3+3\,e^4}\right)^{1/3}}{3\times 3^{2/3}}\right)}-$$

$$\frac{1}{2}\sqrt{\left(\frac{9}{2}-\frac{35\times 2^{2/3}\,e}{3\times 3^{1/3}\left(972\,e+3\,e^2-\sqrt{3}\,\sqrt{314\,928\,e^2-83\,806\,e^3+3\,e^4}\right)^{1/3}}-\frac{2^{1/3}\left(972\,e+3\,e^2-\sqrt{3}\,\sqrt{314\,928\,e^2-83\,806\,e^3+3\,e^4}\right)^{1/3}}{3\times 3^{2/3}}-\frac{-243-\frac{8}{9}\left(-243-2\,e\right)}{4\sqrt{\frac{9}{4}+\frac{35\times 2^{2/3}\,e}{3\times 3^{1/3}\left(972\,e+3\,e^2-\sqrt{3}\,\sqrt{314\,928\,e^2-83\,806\,e^3+3\,e^4}\right)^{1/3}}+\frac{2^{1/3}\left(972\,e+3\,e^2-\sqrt{3}\,\sqrt{314\,928\,e^2-83\,806\,e^3+3\,e^4}\right)^{1/3}}{3\times 3^{2/3}}}}\right)}$$

In[18]:=

```
(*the effect of efficiency e on p*ii*)
pii = a (6 + s) / (6 (2 + s)) /. a → 1
```

Out[18]=

$$\left(\frac{19}{4}+\frac{1}{2}\sqrt{\left(\frac{9}{4}+\frac{35\times 2^{2/3}\,e}{3\times 3^{1/3}\left(972\,e+3\,e^2-\sqrt{3}\,\sqrt{314\,928\,e^2-83\,806\,e^3+3\,e^4}\right)^{1/3}}+\frac{2^{1/3}\left(972\,e+3\,e^2-\sqrt{3}\,\sqrt{314\,928\,e^2-83\,806\,e^3+3\,e^4}\right)^{1/3}}{3\times 3^{2/3}}\right)}-\right.$$

$$\frac{1}{2}\sqrt{\left(\frac{9}{2}-\frac{35\times 2^{2/3}\,e}{3\times 3^{1/3}\left(972\,e+3\,e^2-\sqrt{3}\,\sqrt{314\,928\,e^2-83\,806\,e^3+3\,e^4}\right)^{1/3}}-\frac{2^{1/3}\left(972\,e+3\,e^2-\sqrt{3}\,\sqrt{314\,928\,e^2-83\,806\,e^3+3\,e^4}\right)^{1/3}}{3\times 3^{2/3}}-\frac{-243-\frac{8}{9}\left(-243-2\,e\right)}{4\sqrt{\frac{9}{4}+\frac{35\times 2^{2/3}\,e}{3\times 3^{1/3}\left(972\,e+3\,e^2-\sqrt{3}\,\sqrt{314\,928\,e^2-83\,806\,e^3+3\,e^4}\right)^{1/3}}+\frac{2^{1/3}\left(972\,e+3\,e^2-\sqrt{3}\,\sqrt{314\,928\,e^2-83\,806\,e^3+3\,e^4}\right)^{1/3}}{3\times 3^{2/3}}}}\right)}\Bigg)\Bigg)\Big/$$

$$\Bigg(6\Bigg(\frac{3}{4}+\frac{1}{2}\sqrt{\left(\frac{9}{4}+\frac{35\times 2^{2/3}\,e}{3\times 3^{1/3}\left(972\,e+3\,e^2-\sqrt{3}\,\sqrt{314\,928\,e^2-83\,806\,e^3+3\,e^4}\right)^{1/3}}+\frac{2^{1/3}\left(972\,e+3\,e^2-\sqrt{3}\,\sqrt{314\,928\,e^2-83\,806\,e^3+3\,e^4}\right)^{1/3}}{3\times 3^{2/3}}\right)}-$$

$$\frac{1}{2}\sqrt{\left(\frac{9}{2}-\frac{35\times 2^{2/3}\,e}{3\times 3^{1/3}\left(972\,e+3\,e^2-\sqrt{3}\,\sqrt{314\,928\,e^2-83\,806\,e^3+3\,e^4}\right)^{1/3}}-\frac{2^{1/3}\left(972\,e+3\,e^2-\sqrt{3}\,\sqrt{314\,928\,e^2-83\,806\,e^3+3\,e^4}\right)^{1/3}}{3\times 3^{2/3}}-\frac{-243-\frac{8}{9}\left(-243-2\,e\right)}{4\sqrt{\frac{9}{4}+\frac{35\times 2^{2/3}\,e}{3\times 3^{1/3}\left(972\,e+3\,e^2-\sqrt{3}\,\sqrt{314\,928\,e^2-83\,806\,e^3+3\,e^4}\right)^{1/3}}+\frac{2^{1/3}\left(972\,e+3\,e^2-\sqrt{3}\,\sqrt{314\,928\,e^2-83\,806\,e^3+3\,e^4}\right)^{1/3}}{3\times 3^{2/3}}}}\right)}\Bigg)\Bigg)\Bigg)$$

In[19]:=

```
Plot[pii, {e, 3.6, 3.76}]
```

Out[19]=

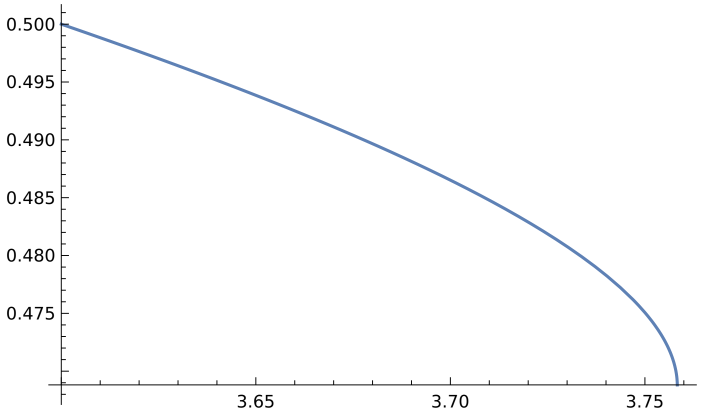


```
(*the effect of efficiency e on p*ij*)
pij = a*(8 - s^2)/(6*(2 + s)) /. a → 1
```

Out[20]=

$$\left(8-\left(-\frac{5}{4}+\frac{1}{2}\sqrt{\left(\frac{9}{4}+\frac{35\times 2^{2/3}\,e}{3\times 3^{1/3}\left(972\,e+3\,e^{2}-\sqrt{3}\,\sqrt{314\,928\,e^{2}-83\,806\,e^{3}+3\,e^{4}}\right)^{1/3}}+\right.}\right.\right.$$

$$\left.\frac{2^{1/3}\left(972\,e+3\,e^{2}-\sqrt{3}\,\sqrt{314\,928\,e^{2}-83\,806\,e^{3}+3\,e^{4}}\right)^{1/3}}{3\times 3^{2/3}}\right)-$$

$$\frac{1}{2}\sqrt{\left(\frac{9}{2}-\frac{35\times 2^{2/3}\,e}{3\times 3^{1/3}\left(972\,e+3\,e^{2}-\sqrt{3}\,\sqrt{314\,928\,e^{2}-83\,806\,e^{3}+3\,e^{4}}\right)^{1/3}}-\right.}$$

$$\frac{2^{1/3}\left(972\,e+3\,e^{2}-\sqrt{3}\,\sqrt{314\,928\,e^{2}-83\,806\,e^{3}+3\,e^{4}}\right)^{1/3}}{3\times 3^{2/3}}-\left(-243-\frac{8}{9}\left(-243-2\,e\right)\right)\Big/$$

$$\left(4\sqrt{\left(\frac{9}{4}+\frac{35\times 2^{2/3}\,e}{3\times 3^{1/3}\left(972\,e+3\,e^{2}-\sqrt{3}\,\sqrt{314\,928\,e^{2}-83\,806\,e^{3}+3\,e^{4}}\right)^{1/3}}+\right.}\right.$$

$$\left.\left.\left.\left.\left.\frac{2^{1/3}\left(972\,e+3\,e^{2}-\sqrt{3}\,\sqrt{314\,928\,e^{2}-83\,806\,e^{3}+3\,e^{4}}\right)^{1/3}}{3\times 3^{2/3}}\right)\right)\right)\right)^{2}\right)\Big/$$

$$\left(6\left(\frac{3}{4}+\frac{1}{2}\sqrt{\left(\frac{9}{4}+\frac{35\times 2^{2/3}\,e}{3\times 3^{1/3}\left(972\,e+3\,e^{2}-\sqrt{3}\,\sqrt{314\,928\,e^{2}-83\,806\,e^{3}+3\,e^{4}}\right)^{1/3}}+\right.}\right.\right.$$

$$\left.\frac{2^{1/3}\left(972\,e+3\,e^2-\sqrt{3}\ \sqrt{314\,928\,e^2-83\,806\,e^3+3\,e^4}\right)^{1/3}}{3\times 3^{2/3}}\right)-$$

$$\frac{1}{2}\sqrt{\left(\frac{9}{2}-\frac{35\times 2^{2/3}\,e}{3\times 3^{1/3}\left(972\,e+3\,e^2-\sqrt{3}\ \sqrt{314\,928\,e^2-83\,806\,e^3+3\,e^4}\right)^{1/3}}-\right.}$$

$$\frac{2^{1/3}\left(972\,e+3\,e^2-\sqrt{3}\ \sqrt{314\,928\,e^2-83\,806\,e^3+3\,e^4}\right)^{1/3}}{3\times 3^{2/3}}-$$

$$\left.\left.\left.\frac{-243-\frac{8}{9}\left(-243-2\,e\right)}{4\sqrt{\frac{9}{4}+\frac{35\times 2^{2/3}\,e}{3\times 3^{1/3}\left(972\,e+3\,e^2-\sqrt{3}\ \sqrt{314\,928\,e^2-83\,806\,e^3+3\,e^4}\right)^{1/3}}+\frac{2^{1/3}\left(972\,e+3\,e^2-\sqrt{3}\ \sqrt{314\,928\,e^2-83\,806\,e^3+3\,e^4}\right)^{1/3}}{3\times 3^{2/3}}}}\right)\right)\right)$$

In[21]:=

```
Plot[pij, {e, 3.6, 3.76}]
```

Out[21]=

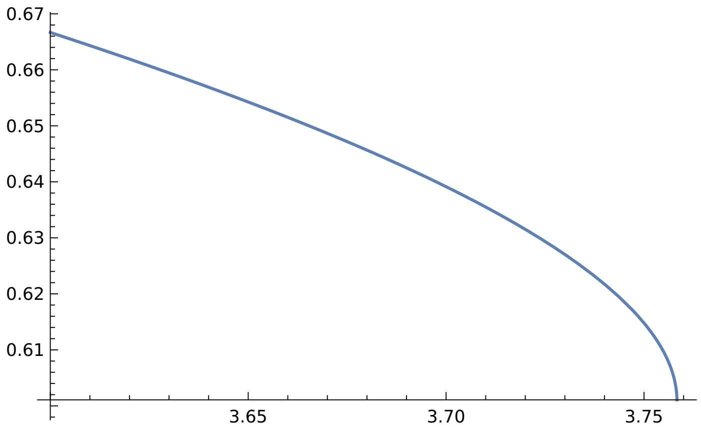

In[22]:=

```
(*the effect of efficiency e on f*)
f = (2 - s) * a / 6 /. a → 1
```

Out[22]=

$$\frac{1}{6}\left(\frac{13}{4}-\frac{1}{2}\sqrt{\left(\frac{9}{4}+\frac{35\times 2^{2/3}\,e}{3\times 3^{1/3}\left(972\,e+3\,e^{2}-\sqrt{3}\,\sqrt{314\,928\,e^{2}-83\,806\,e^{3}+3\,e^{4}}\right)^{1/3}}+\frac{2^{1/3}\left(972\,e+3\,e^{2}-\sqrt{3}\,\sqrt{314\,928\,e^{2}-83\,806\,e^{3}+3\,e^{4}}\right)^{1/3}}{3\times 3^{2/3}}\right)}+\frac{1}{2}\sqrt{\left(\frac{9}{2}-\frac{35\times 2^{2/3}\,e}{3\times 3^{1/3}\left(972\,e+3\,e^{2}-\sqrt{3}\,\sqrt{314\,928\,e^{2}-83\,806\,e^{3}+3\,e^{4}}\right)^{1/3}}-\frac{2^{1/3}\left(972\,e+3\,e^{2}-\sqrt{3}\,\sqrt{314\,928\,e^{2}-83\,806\,e^{3}+3\,e^{4}}\right)^{1/3}}{3\times 3^{2/3}}-\frac{-243-\frac{8}{9}\left(-243-2\,e\right)}{4\sqrt{\frac{9}{4}+\frac{35\times 2^{2/3}\,e}{3\times 3^{1/3}\left(972\,e+3\,e^{2}-\sqrt{3}\,\sqrt{314\,928\,e^{2}-83\,806\,e^{3}+3\,e^{4}}\right)^{1/3}}+\frac{2^{1/3}\left(972\,e+3\,e^{2}-\sqrt{3}\,\sqrt{314\,928\,e^{2}-83\,806\,e^{3}+3\,e^{4}}\right)^{1/3}}{3\times 3^{2/3}}}}\right)}\right)$$

In[23]:=

```
Plot[{f}, {e, 3.6, 3.76}]
```

Out[23]=

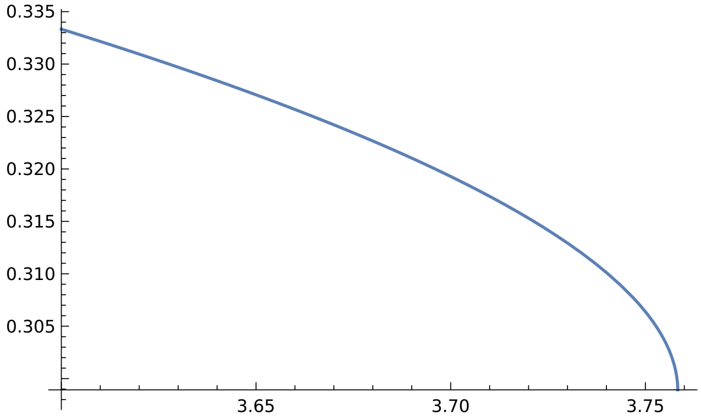

In[24]:=

```
(*the effect of efficiency e on q*ii*)
qii = ((2 - s) * a + s * f) / (4 - s ^ 2) /. a → 1
```

Out[24]=

$$\left(\frac{13}{4}-\frac{1}{2}\sqrt{\left(\frac{9}{4}+\frac{35\times 2^{2/3}\,e}{3\times 3^{1/3}\left(972\,e+3\,e^2-\sqrt{3}\,\sqrt{314\,928\,e^2-83\,806\,e^3+3\,e^4}\right)^{1/3}}+\frac{2^{1/3}\left(972\,e+3\,e^2-\sqrt{3}\,\sqrt{314\,928\,e^2-83\,806\,e^3+3\,e^4}\right)^{1/3}}{3\times 3^{2/3}}\right)}+\right.$$

$$\frac{1}{2}\sqrt{\left(\frac{9}{2}-\frac{35\times 2^{2/3}\,e}{3\times 3^{1/3}\left(972\,e+3\,e^2-\sqrt{3}\,\sqrt{314\,928\,e^2-83\,806\,e^3+3\,e^4}\right)^{1/3}}-\frac{2^{1/3}\left(972\,e+3\,e^2-\sqrt{3}\,\sqrt{314\,928\,e^2-83\,806\,e^3+3\,e^4}\right)^{1/3}}{3\times 3^{2/3}}-\frac{-243-\frac{8}{9}\left(-243-2\,e\right)}{4\sqrt{\frac{9}{4}+\frac{35\times 2^{2/3}\,e}{3\times 3^{1/3}\left(972\,e+3\,e^2-\sqrt{3}\,\sqrt{314\,928\,e^2-83\,806\,e^3+3\,e^4}\right)^{1/3}}+\frac{2^{1/3}\left(972\,e+3\,e^2-\sqrt{3}\,\sqrt{314\,928\,e^2-83\,806\,e^3+3\,e^4}\right)^{1/3}}{3\times 3^{2/3}}}}\right)}+$$

$$\frac{1}{6}\left(-\frac{5}{4}+\frac{1}{2}\sqrt{\left(\frac{9}{4}+\frac{35\times 2^{2/3}\,e}{3\times 3^{1/3}\left(972\,e+3\,e^2-\sqrt{3}\,\sqrt{314\,928\,e^2-83\,806\,e^3+3\,e^4}\right)^{1/3}}+\frac{2^{1/3}\left(972\,e+3\,e^2-\sqrt{3}\,\sqrt{314\,928\,e^2-83\,806\,e^3+3\,e^4}\right)^{1/3}}{3\times 3^{2/3}}\right)}-\right.$$

$$\frac{1}{2}\sqrt{\left(\frac{9}{2}-\frac{35\times 2^{2/3}\,e}{3\times 3^{1/3}\left(972\,e+3\,e^{2}-\sqrt{3}\,\sqrt{314\,928\,e^{2}-83\,806\,e^{3}+3\,e^{4}}\right)^{1/3}}-\right.}$$

$$\frac{2^{1/3}\left(972\,e+3\,e^{2}-\sqrt{3}\,\sqrt{314\,928\,e^{2}-83\,806\,e^{3}+3\,e^{4}}\right)^{1/3}}{3\times 3^{2/3}}-$$

$$\frac{-243-\frac{8}{9}\left(-243-2\,e\right)}{4\sqrt{\frac{9}{4}+\frac{35\times 2^{2/3}\,e}{3\times 3^{1/3}\left(972\,e+3\,e^{2}-\sqrt{3}\,\sqrt{314\,928\,e^{2}-83\,806\,e^{3}+3\,e^{4}}\right)^{1/3}}+\frac{2^{1/3}\left(972\,e+3\,e^{2}-\sqrt{3}\,\sqrt{314\,928\,e^{2}-83\,806\,e^{3}+3\,e^{4}}\right)^{1/3}}{3\times 3^{2/3}}}}\Bigg)\Bigg)$$

$$\Bigg(\frac{13}{4}-\frac{1}{2}\sqrt{\left(\frac{9}{4}+\frac{35\times 2^{2/3}\,e}{3\times 3^{1/3}\left(972\,e+3\,e^{2}-\sqrt{3}\,\sqrt{314\,928\,e^{2}-83\,806\,e^{3}+3\,e^{4}}\right)^{1/3}}+\right.}$$

$$\left.\frac{2^{1/3}\left(972\,e+3\,e^{2}-\sqrt{3}\,\sqrt{314\,928\,e^{2}-83\,806\,e^{3}+3\,e^{4}}\right)^{1/3}}{3\times 3^{2/3}}\right)+$$

$$\frac{1}{2}\sqrt{\left(\frac{9}{2}-\frac{35\times 2^{2/3}\,e}{3\times 3^{1/3}\left(972\,e+3\,e^{2}-\sqrt{3}\,\sqrt{314\,928\,e^{2}-83\,806\,e^{3}+3\,e^{4}}\right)^{1/3}}-\right.}$$

$$\frac{2^{1/3}\left(972\,e+3\,e^{2}-\sqrt{3}\,\sqrt{314\,928\,e^{2}-83\,806\,e^{3}+3\,e^{4}}\right)^{1/3}}{3\times 3^{2/3}}-$$

$$\frac{-243-\frac{8}{9}\left(-243-2\,e\right)}{4\sqrt{\frac{9}{4}+\frac{35\times 2^{2/3}\,e}{3\times 3^{1/3}\left(972\,e+3\,e^{2}-\sqrt{3}\,\sqrt{314\,928\,e^{2}-83\,806\,e^{3}+3\,e^{4}}\right)^{1/3}}+\frac{2^{1/3}\left(972\,e+3\,e^{2}-\sqrt{3}\,\sqrt{314\,928\,e^{2}-83\,806\,e^{3}+3\,e^{4}}\right)^{1/3}}{3\times 3^{2/3}}}}\Bigg)\Bigg)\Bigg)$$

$$\Bigg(4 - \Bigg(-\frac{5}{4} + \frac{1}{2}\sqrt{\Bigg(\frac{9}{4} + \frac{35 \times 2^{2/3}\, e}{3 \times 3^{1/3} \left(972\, e + 3\, e^2 - \sqrt{3}\ \sqrt{314\,928\, e^2 - 83\,806\, e^3 + 3\, e^4}\right)^{1/3}} +}$$

$$\frac{2^{1/3} \left(972\, e + 3\, e^2 - \sqrt{3}\ \sqrt{314\,928\, e^2 - 83\,806\, e^3 + 3\, e^4}\right)^{1/3}}{3 \times 3^{2/3}}\Bigg) -$$

$$\frac{1}{2}\sqrt{\Bigg(\frac{9}{2} - \frac{35 \times 2^{2/3}\, e}{3 \times 3^{1/3} \left(972\, e + 3\, e^2 - \sqrt{3}\ \sqrt{314\,928\, e^2 - 83\,806\, e^3 + 3\, e^4}\right)^{1/3}} -}$$

$$\frac{2^{1/3} \left(972\, e + 3\, e^2 - \sqrt{3}\ \sqrt{314\,928\, e^2 - 83\,806\, e^3 + 3\, e^4}\right)^{1/3}}{3 \times 3^{2/3}} - \left(-243 - \frac{8}{9}\left(-243 - 2\, e\right)\right) \Big/$$

$$\Bigg(4\sqrt{\Bigg(\frac{9}{4} + \frac{35 \times 2^{2/3}\, e}{3 \times 3^{1/3} \left(972\, e + 3\, e^2 - \sqrt{3}\ \sqrt{314\,928\, e^2 - 83\,806\, e^3 + 3\, e^4}\right)^{1/3}} +}$$

$$\frac{2^{1/3} \left(972\, e + 3\, e^2 - \sqrt{3}\ \sqrt{314\,928\, e^2 - 83\,806\, e^3 + 3\, e^4}\right)^{1/3}}{3 \times 3^{2/3}}\Bigg)\Bigg)\Bigg)\Bigg)^2\Bigg)$$

In[25]:=

```
Plot[qii, {e, 3.6, 3.76}]
```

Out[25]=

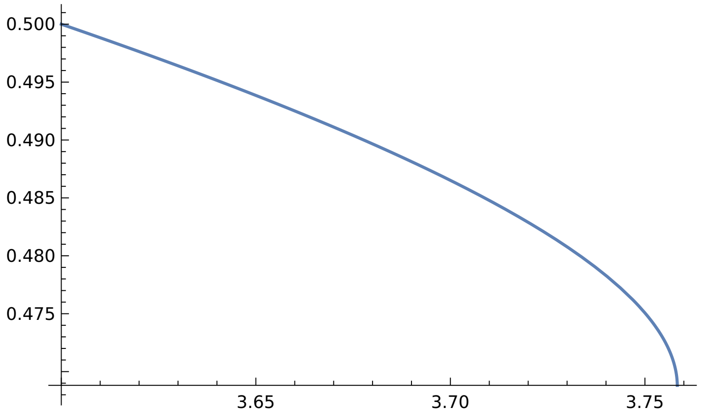

In[26]:=

```
(*the effect of efficiency e on q*ij*)
qij = ((2 - s) * a - s * 2) / (4 - s ^ 2) /. a → 1
```

Out[26]=

$$\Bigg(\frac{13}{4} - \frac{1}{2}\sqrt{\Bigg(\frac{9}{4} + \frac{35 \times 2^{2/3}\, e}{3 \times 3^{1/3} \left(972\, e + 3\, e^2 - \sqrt{3}\ \sqrt{314\,928\, e^2 - 83\,806\, e^3 + 3\, e^4}\right)^{1/3}} +}$$

$$\left.\frac{2^{1/3}\left(972\,e+3\,e^2-\sqrt{3}\;\sqrt{314\,928\,e^2-83\,806\,e^3+3\,e^4}\right)^{1/3}}{3\times 3^{2/3}}\right)+$$

$$\frac{1}{2}\sqrt{\left(\frac{9}{2}-\frac{35\times 2^{2/3}\,e}{3\times 3^{1/3}\left(972\,e+3\,e^2-\sqrt{3}\;\sqrt{314\,928\,e^2-83\,806\,e^3+3\,e^4}\right)^{1/3}}-\right.}$$

$$\frac{2^{1/3}\left(972\,e+3\,e^2-\sqrt{3}\;\sqrt{314\,928\,e^2-83\,806\,e^3+3\,e^4}\right)^{1/3}}{3\times 3^{2/3}}-$$

$$\left.\frac{-243-\frac{8}{9}\left(-243-2\,e\right)}{4\sqrt{\frac{9}{4}+\frac{35\times 2^{2/3}\,e}{3\times 3^{1/3}\left(972\,e+3\,e^2-\sqrt{3}\;\sqrt{314\,928\,e^2-83\,806\,e^3+3\,e^4}\right)^{1/3}}+\frac{2^{1/3}\left(972\,e+3\,e^2-\sqrt{3}\;\sqrt{314\,928\,e^2-83\,806\,e^3+3\,e^4}\right)^{1/3}}{3\times 3^{2/3}}}}\right)-$$

$$2\left(-\frac{5}{4}+\frac{1}{2}\sqrt{\left(\frac{9}{4}+\frac{35\times 2^{2/3}\,e}{3\times 3^{1/3}\left(972\,e+3\,e^2-\sqrt{3}\;\sqrt{314\,928\,e^2-83\,806\,e^3+3\,e^4}\right)^{1/3}}+\right.}\right.$$

$$\left.\frac{2^{1/3}\left(972\,e+3\,e^2-\sqrt{3}\;\sqrt{314\,928\,e^2-83\,806\,e^3+3\,e^4}\right)^{1/3}}{3\times 3^{2/3}}\right)-$$

$$\frac{1}{2}\sqrt{\left(\frac{9}{2}-\frac{35\times 2^{2/3}\,e}{3\times 3^{1/3}\left(972\,e+3\,e^2-\sqrt{3}\;\sqrt{314\,928\,e^2-83\,806\,e^3+3\,e^4}\right)^{1/3}}-\right.}$$

$$\frac{2^{1/3}\left(972\,e+3\,e^2-\sqrt{3}\;\sqrt{314\,928\,e^2-83\,806\,e^3+3\,e^4}\right)^{1/3}}{3\times 3^{2/3}}-$$

$$\frac{-243-\frac{8}{9}\left(-243-2\,e\right)}{4\sqrt{\frac{9}{4}+\frac{35\times 2^{2/3}\,e}{3\times 3^{1/3}\left(972\,e+3\,e^{2}-\sqrt{3}\,\sqrt{314\,928\,e^{2}-83\,806\,e^{3}+3\,e^{4}}\right)^{1/3}}+\frac{2^{1/3}\left(972\,e+3\,e^{2}-\sqrt{3}\,\sqrt{314\,928\,e^{2}-83\,806\,e^{3}+3\,e^{4}}\right)^{1/3}}{3\times 3^{2/3}}}}\Bigg)\Bigg)\Bigg)$$

$$\Bigg(4-\Bigg(-\frac{5}{4}+\frac{1}{2}\sqrt{\Bigg(\frac{9}{4}+\frac{35\times 2^{2/3}\,e}{3\times 3^{1/3}\left(972\,e+3\,e^{2}-\sqrt{3}\,\sqrt{314\,928\,e^{2}-83\,806\,e^{3}+3\,e^{4}}\right)^{1/3}}+\frac{2^{1/3}\left(972\,e+3\,e^{2}-\sqrt{3}\,\sqrt{314\,928\,e^{2}-83\,806\,e^{3}+3\,e^{4}}\right)^{1/3}}{3\times 3^{2/3}}\Bigg)}-$$

$$\frac{1}{2}\sqrt{\Bigg(\frac{9}{2}-\frac{35\times 2^{2/3}\,e}{3\times 3^{1/3}\left(972\,e+3\,e^{2}-\sqrt{3}\,\sqrt{314\,928\,e^{2}-83\,806\,e^{3}+3\,e^{4}}\right)^{1/3}}-\frac{2^{1/3}\left(972\,e+3\,e^{2}-\sqrt{3}\,\sqrt{314\,928\,e^{2}-83\,806\,e^{3}+3\,e^{4}}\right)^{1/3}}{3\times 3^{2/3}}-\left(-243-\frac{8}{9}\left(-243-2\,e\right)\right)\Big/}$$

$$\Bigg(4\sqrt{\Bigg(\frac{9}{4}+\frac{35\times 2^{2/3}\,e}{3\times 3^{1/3}\left(972\,e+3\,e^{2}-\sqrt{3}\,\sqrt{314\,928\,e^{2}-83\,806\,e^{3}+3\,e^{4}}\right)^{1/3}}+\frac{2^{1/3}\left(972\,e+3\,e^{2}-\sqrt{3}\,\sqrt{314\,928\,e^{2}-83\,806\,e^{3}+3\,e^{4}}\right)^{1/3}}{3\times 3^{2/3}}\Bigg)}\Bigg)\Bigg)\Bigg)^{2}\Bigg)$$

In[27]:=

```
Plot[qij, {e, 3.6, 3.76}]
```

Out[27]=

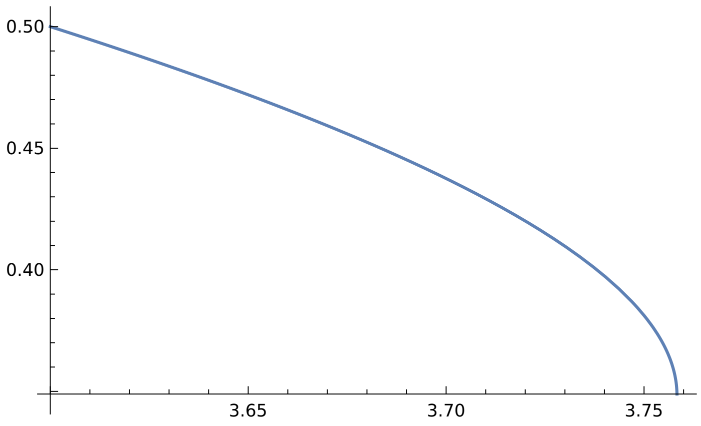

In[28]:=

```
(*the effect of efficiency e on the level of trade q*ij/qii*)
Plot[{qij / qii}, {e, 3.6, 3.76}]
```

Out[28]=

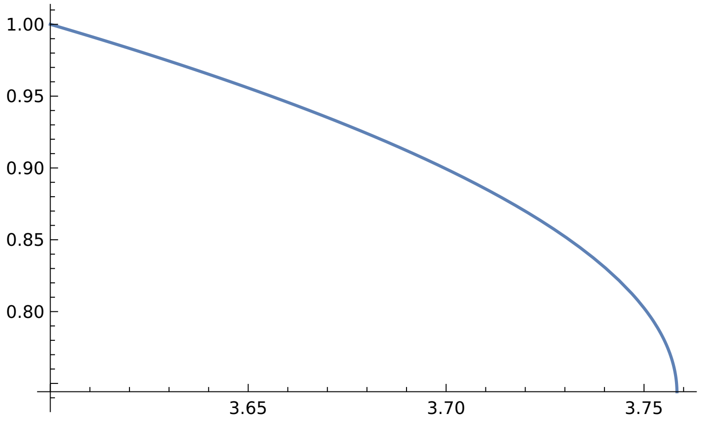

In[29]:=

```
(*the effect of efficiency e on π*i*)
profit = pii * qii + pij * qij - f * qij - (1 / (2 e)) * d ^ 2 /. a → 1
```

Out[29]=

$$-\frac{\left(\frac{9}{4}-\frac{1}{2}\sqrt{\frac{9}{4}+\cdots 1\cdots+\cdots 1\cdots}+\frac{1}{2}\sqrt{\cdots 1\cdots}\right)^2}{2\,e}-\frac{\cdots 1\cdots}{6\ \cdots 1\cdots}+\cdots 1\cdots+\frac{\left(\frac{19}{4}+\frac{1}{2}\cdots 1\cdots-\frac{1}{2}\sqrt{\frac{9}{2}-\frac{\cdots 1\cdots}{\cdots 1\cdots}-\cdots 1\cdots-\frac{-243-\cdots 1\cdots}{4\sqrt{\cdots 1\cdots}}}\right)\cdots 1\cdots}{6\left(\frac{3}{4}+\frac{1}{2}\sqrt{\frac{9}{4}+\cdots 1\cdots+\cdots 1\cdots}-\frac{1}{2}\sqrt{\cdots 1\cdots}\right)\left(4-\left(-\frac{5}{4}+\frac{1}{2}\cdots 1\cdots-\frac{1}{2}\sqrt{\cdots 1\cdots}\right)^2\right)}$$

Full expression not available (original memory size: 0.2 MB)

In[31]:=

```
Plot[profit, {e, 3.6, 3.76}]
```

Out[31]=

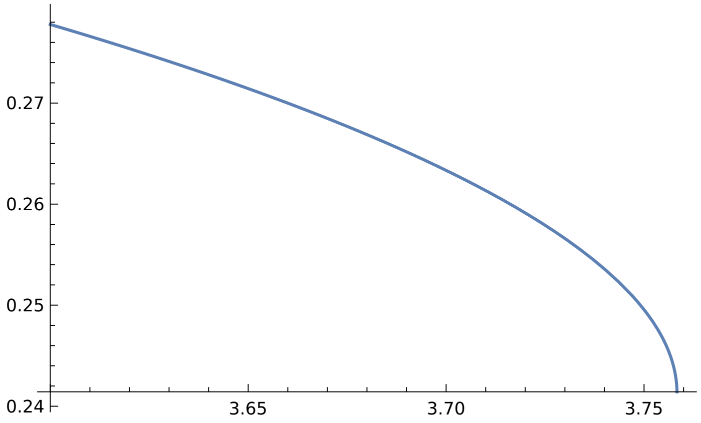

Supplement: S1 File — (ZIP) [file pone.0273904.s001.zip › Supporting Information/PLOS ONE_minimal underlying data set.pdf]
